# Measuring complex constructs in large-scale text with computational social mixed methods

**DOI:** 10.3758/s13428-026-03046-5

**Published:** 2026-07-21

**Authors:** Alina Herderich, Jana Lasser, Mirta Galesic, Segun Aroyehun, David Garcia, Joshua Garland

**Affiliations:** 1 IDea_Lab, University of Graz, Leechgasse 34, 8010 Graz, Austria; 2 Department of Computer Science and Biomedical Engineering, Graz University of Technology, Graz, Austria; 3 Complexity Science Hub Vienna, Vienna, Austria; 4 Santa Fe Institute, Santa Fe, NM USA; 5 Vermont Complex Systems Center, University of Vermont, Burlington, VT USA; 6 Department of Politics and Public Administration, University of Konstanz, Konstanz, Germany; 7 Center on Information and Narrative Complexity, Arizona State University, Tempe, AZ USA

**Keywords:** Text-as-data, Classification, Machine learning, Computational social science, Mixed methods

## Abstract

A growing convergence between social science and machine learning enables, in principle, large-scale analyses of complex social phenomena through text. Yet, approaches leveraging supervised text classification based on human-annotated data for statistical analysis often treat conceptual validity and technical performance as separate challenges, impairing measurement quality. We provide guidelines to bridge this gap in what we call *computational social mixed methods pipelines* across three stages: data annotation, model training, and statistical analysis. Building on best practices and our own methodological innovations, such as “Iterative Annotation” and “Training on Confident Examples”, we address recurring pitfalls like unbalanced training data or stagnant model performance. We also discuss when large language models constitute a viable alternative to transformer-based classifiers. Using a case study on countering online hate, we illustrate how consequently integrating social science and machine learning expertise improves the validity and comparability of computational social science.

Over the last decade, computational methods have pervaded nearly all areas of research, from the natural and engineering sciences to the social sciences, including psychology (Wang et al., 2023). Integrating computational methods in the social science toolkit has several major advantages. For example, it has introduced the social sciences as “predictive sciences” where accurate predictions are employed as a means to achieve robust explanations instead of vice versa (Kleinberg, Lakkaraju, Leskovec, Ludwig, & Mullainathan, 2017; Salganik et al., 2020; Yarkoni & Westfall, 2017). It also allows the testing of hypotheses at scale (e.g., nature versus nurture in emotions; Lindquist, Jackson, Leshin, Satpute, & Gendron, 2022), including the rethinking of individual-level concepts in larger societal contexts (e.g., collective emotions; Garcia & Rimé, 2019; Goldenberg, Garcia, Halperin, & Gross, 2020).

Applying computational methods to social science questions—also termed computational social science (Lazer et al., 2009)—has allowed us to tackle questions that are central to the human nature and our society today (e.g., misinformation, political communication, and societal discourse; Argyle et al., 2023; Bak-Coleman et al., 2022; Bellovary, Young, & Goldenberg, 2021; Brady, Wills, Jost, Tucker, & Bavel, 2017; Grinberg, Joseph, Friedland, Swire-Thompson, & Lazer, 2019; Lasser et al., 2023; Lasser et al., 2025; McLoughlin & Brady, 2023; Osmunden, Bor, Vahlstrup, Bechmann, & Petersen, 2021; Robertson et al., 2023; Schöne, Garcia, Parkinson, & Goldenberg, 2023), especially through making accessible both large data sets, and methods to process them (Feuerriegel et al., 2025). Such data often includes naturally observed human behavior (Salganik et al., 2020), complementing the restricted ecological validity of small-scale experiments, and therefore providing a valuable complementary perspective on both existing and emerging phenomena.

## What are computational social mixed methods pipelines?

In computational social science, a common task is the quantification of a specific phenomenon in text, combining human annotation and machine learning classifiers to scale up the measurement, which can then be used in statistical modeling for inference or prediction. This text is often large in scale, meaning that the manual inspection of the entire corpus is infeasible. The phenomena, on the other hand, are complex in that they are latent constructs with multiple conceptualizations and operationalizations. After the early euphoria about combining human annotation, machine learning, and statistical analysis to study phenomena as they naturally occur, both computational and social science researchers are starting to recognize its limitations. The approach spanning multiple methodologies from previously unrelated fields makes it challenging to execute well, since each of these methodologies is backed up by a long disciplinary history and requires several years of training to master. For example, from a social science perspective, predictive performance of machine learning classifiers is only a weak proof for validity (Baden, Pipal, Schoonvelde, & van der Velden, 2022), while from a computational perspective, models in social science applications might lack sophistication due to the fast progression of the field.

We use the term computational social mixed methods to describe a pipeline that includes social science and computational, as well as qualitative and quantitative approaches. One instance of such a *computational social mixed methods pipeline* can consist of data annotation, machine learning classification, and statistical analysis, and our guidelines explain how to achieve a well-rounded pipeline from start to finish.

Our contributions are twofold: First, we cover the entire pipeline instead of focusing on parts contributed by individual disciplines and demonstrate how synergies can emerge throughout. Best practices for annotation, classification, and analysis exist, but are scattered across the social science and machine learning literature (e.g., see Barberá, Boydstun, Linn, McMahon, & Nagler, 2021 with a focus on the curation of training data; Maier et al., 2018; Waldherr et al., 2019 with a focus on classification scheme development supported by unsupervised machine learning; Baden, Kligler-Vilenchik, & Yarchi 2020 with a focus on topic modeling and content analysis; and Feuerriegel et al., 2025 and Wankmüller, 2024 with a general review of transformer models and/or NLP methods).

Second, we provide concrete advice on overcoming common challenges in implementing computational social mixed methods pipelines. To this end, we review the literature for best practices where possible, but also introduce new solutions where not readily available. Published work leveraging computational social mixed methods has to pass certain quality criteria, but solutions for failing those criteria are rarely discussed. For example, when is low interrater reliability a concern, and how can we address it? Computational social mixed methods pipelines have multiple points of failure, increasing their risk of landing in the metaphorical file drawer.

**Fig. 1:**
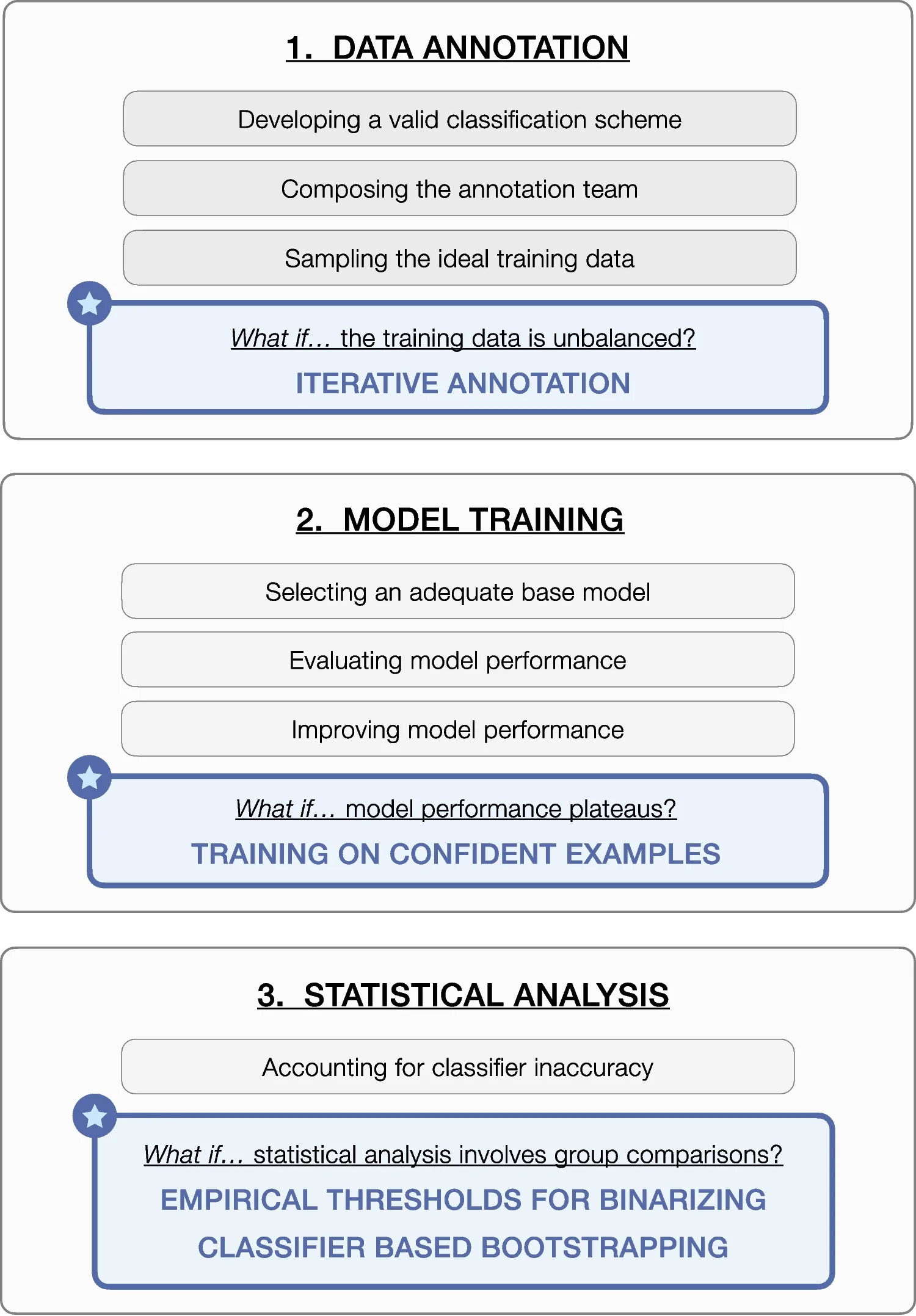
Schematic depiction of a computational social mixed methods pipeline. *Note.* This pipeline focuses specifically on (1) data annotation, (2) machine learning classification, and (3) statistical analysis. For each step in the pipeline, we review best practices from the literature. We further highlight some common points of failure and specific challenges for which we propose original solutions, here marked with a star

Our guidelines focus on computational social mixed methods pipelines, including (1) data annotation, (2) machine learning classifier training, and (3) statistical analysis as illustrated in Fig. 1. More specifically, in the part on data annotation, we cover the data-driven development of a classification scheme with Grounded Theory (Glaser & Strauss, 2010), and discuss the composition of the annotation team and training data. In the second part on model training, we describe how to select an adequate base model for machine classification, and evaluate and improve its performance including a discussion on large language models (LLMs) versus traditional transformer models (i.e., embedding models with a classification head) as classifiers. Finally, in the part on statistical analysis, we focus on regression analysis and group comparisons, and demonstrate how those can be made robust to the inaccuracies of the machine learning classifier.

We further provide original solutions for selected, but common challenges in computational social mixed methods pipelines (see Fig. 1 for details): (I) When sampling the training data, unbalanced data can hinder the machine learning classifiers from picking up underrepresented classes. We introduce a concept termed “Iterative Annotation” to counteract unbalanced training data that can be applied at the stage of data annotation instead of post hoc when data has already been collected. (II) While improving the classification model, performance can plateau despite sufficient training data. We introduce a training strategy that exploits the most informative data points in the training data, called “Training on Confident Examples” showing that less data of higher quality can be superior to more data of lesser quality. (III) When accounting for classifier inaccuracy during statistical analysis involving group comparisons, choosing random classifier thresholds to binarize classes can skew the results. Instead of choosing arbitrarily, we describe an empirical method to select a classification threshold to assign samples to groups that are maximally distinct. (IV) Even with empirically selected thresholds, the classifier still has a remaining error rate, effectively assigning samples to inadequate groups. We explain how inaccuracies in the classification model can be mitigated in the statistical analysis by bootstrapping confidence intervals based on the classifier’s false-positive and false-negative rates.

We demonstrate best practices and new solutions on a working example from our own research, which we introduce next.

**Table 1:** Considerations and best practices in computational social mixed methods pipelines

How to ...	Best practices
Data Annotation	
... specify the classification scheme?	- theory-driven if viable classifications or evidence exist and apply to the sample
	- data-driven with Grounded Theory or unsupervised machine learning
... compose the annotation team?	- non-experts for tasks with clear linguistic markers, experts for latent concepts
	- balanced across relevant personal context variables to avoid bias
	- more annotators if the concept is inter-subjective and context-dependent
	- for simple tasks, consider using LLMs as annotators
... sample the training data?	- more labels if the concept is inter-subjective and context-dependent
	- track IRR during annotation and mitigate insufficient IRR later
	- sample from bins of confounding variables in the corpus
	- consider techniques from data perspectivism to create a held-out test set
	- **use “Iterative Annotation” to counteract unbalanced training data**
Model Training	
... select an adequate base model?	- only use LLMs for simple labeling instructions and consider API costs
... evaluate model performance?	- accuracy, F1 score, receiver operating characteristic (ROC), area under the curve (AUC), confusion matrix as common performance indicators, n-fold cross-validation
... improve model performance?	- masked language modeling (MLM), hyperparameter tuning, early stopping
	- **“Training on Confident Examples” if model performance plateaus**
Statistical Analysis	
... account for classifier inaccuracy?	- for regression tasks: regression calibration, multiple overimputation, pseudo-likelihood modeling, maximum likelihood adjustment, design-based supervised learning (DSL), prediction powered inference (PPI)
	- for group comparisons: **tuning classifier thresholds against human annotation**, **bootstrapping confidence intervals based on false positives/negatives**

*Note.* IRR = interrater reliability, LLM = large language model. Text in bold indicates the proposed solutions to common points of failure in computational social mixed-methods pipelines. See also Fig. 1

## Working example: collective moderation of hateful speech in online spaces

In order to protect civil deliberation (Friess, Ziegele, & Heinbach, 2021) on social media platforms as a central means of political communication (Gilardi, Gessler, Kubli, & Müller, 2022; Newman, Fletcher, Eddy, Robertson, & Nielsen, 2023; Siegel, 2020), we need to find ways to moderate hate in online political discussions. Content moderation by platforms has been controversial, because it poses questions of authority over truth and freedom of expression (Hangartner et al., 2021). An inclusive and scalable form of collective civic moderation recently evolving is counter speech (Benesch, Ruths, Dillon, Saleem, & Wright, 2016; Buerger, 2021; Mathew et al., 2019).

In the work that serves as an example for our guidelines (Lasser et al., 2025), we define counter speech as the attempt to counter hateful comments with the goal of steering a discussion toward a more civil and fact-based direction. We investigated effective strategies for collective counter speech (Garland, Ghazi-Zahedi, Young, Hébert-Dufresne, & Galesic, 2020), analyzing a large corpus of over 130,000 political discussions on German Twitter from 2015 to 2018 under posts of prominent news outlets, politicians, and public figures. We investigated how counter speech is expressed in natural conversations, and asked, for example, whether different types of counter speech strategies (e.g., providing facts or raising opinions) are more or less effective in reducing hate speech in the remainder of the discussion. Up until today, research on counter speech has mainly focused on qualitative studies (Benesch et al., 2016; Buerger, 2021; Friess et al., 2021; Keller & Askanius, 2020), or experiments (Álvarez-Benjumea & Winter, 2018; Hangartner et al., 2021; Munger, 2017), both being restricted to comparatively small samples. In our analysis, we use matching and time-series analysis to assess the effectiveness of counter-speech strategies at the level of individual tweet pairs, entire discussions, and over days. We find, among others, that simple opinions without insults are most effective in reducing hate over different time scales.

## How to use our guidelines

Our guidelines are structured along the considerations listed in Fig. 1. In each section, we first describe the purpose of this stage in the pipeline. We then outline potential challenges arising in this step. We review existing best practices from the literature where possible. For selected challenges (see Fig. 1), we introduce novel solutions and illustrate them with an example from our own work on hate and counter speech online (see previous section on “Collective moderation of hateful speech in online spaces”). Table 1 summarizes necessary considerations with their respective best practices, including novel solutions along the pipeline.

In our guidelines, we provide detailed, hands-on advice on integrating perspectives from both computational and social sciences to increase methodological rigor of research designs using human data annotation and automatic text classification. On the one hand, our aim is to raise the confidence of researchers trained in the social sciences to implement state-of-the-art machine learning classification for statistical analysis by providing solutions for common points of failure in such pipelines. On the other hand, we aim to offer guidance for researchers trained in computational methods who wish for a more thorough integration of social science techniques and theory in model training, in particular with respect to developing valid classification schemes and the data annotation process. Our working example illustrates a project with close collaboration from both social science and computational scholars that integrates best practices from both fields. Our guidelines are accompanied by tutorial-style code and data accessible under https://github.com/Hai-Lina/computational-social-mixed-methods-pipelines.

## Data annotation

### Developing a valid classification scheme

#### Purpose and challenges

The goal of developing a classification scheme is to define the concepts the research engages with and how they can be recognized in text, that is, to make explicit the conceptualization, translation, and formalization of a problem described in natural language into quantitative indicators (Kang, 2023).

When developing classification schemes as the basis for machine learning classifiers, one often faces a trade-off between the validity and reliability of construct measurement, that is, simple annotation rules may be reliably picked up by classifiers, but not valid, and vice versa (Feuerriegel et al., 2025). Psychological and sociological constructs are broad theoretical concepts that are only probabilistically related to observable indicators, and different constructs can be expressed more or less directly in language. While humans are able to interpret text context-dependent and recognize the latent meaning of language, machine learning classifiers will struggle to pick up the rules humans implicitly apply. For example, hate speech covers a variety of behaviors that are reflected more or less explicitly in text: Insults and threats are most often characterized by distinct phrasings, however, stereotyping requires knowledge about societal groups and their cultural context (Bakalis, 2015; Blaya, 2019; Google, 2024; Meta, 2024; Weber, 2009; X, 2024).

Even if a data set exists that reliably identifies a construct in text with respect to human perception, there are limits to what machine learning classifiers can learn from that data. In machine learning, models are usually incrementally improved upon a fixed data set (the “ground truth” or “gold standard”) with respect to a single numerical indicator (e.g., F1 score, see also section “Evaluating model performance”), which is easier the narrower the concept is (Liang et al., 2022). Replication studies on prominent benchmark data sets (e.g., the “20 newsgroups” data set) show that labels by majority vote are often impossible to replicate due to substantial variation among raters (Wong, Paritosh, & Bollacker, 2022), which raises the question whether such a “ground truth” exists.

Furthermore, when disconnected from theory, machine learning models have limited usefulness for social science applications. For example, a very prominent data set for the advancement and testing of models to detect discrete emotions in text includes eleven emotion classes (e.g., “anger,” but also “anticipation”) without justifying the choice of these classes (Mohammad & Kiritchenko, 2018), although the number, type and nature of emotions is still heavily debated within psychology itself (Cowen & Keltner, 2021; Paletz et al., 2023; Russell, 2014). In light of machine learning lacking human intuition (e.g., the inability to reason about mental states; Sap, LeBras, Fried, & Choi, 2023), it is essential to establish a good foundation of such models by developing theoretically grounded classification schemes. This problem is exacerbated in the era of LLM text classification, where researchers rely on surface-level definitions and zero-shot classification, leading to biased estimates in statistical analyses and making the findings hard, if not impossible, to interpret (Halterman & Keith, 2025).

#### Best practices

There are two standard routes to developing classification schemes for machine learning in the social sciences: a theory-driven approach, in which researchers form classes based on existing social scientific theory; and a data-driven approach, in which researchers form classes based on data. In a data-driven approach, researchers develop classes bottom-up by qualitatively inspecting their data. The approach to take mainly depends on the availability of theoretical conceptualizations in the literature and on whether existing conceptualizations map onto the research question at hand. Both approaches require establishing a solid working definition of the constructs of interest beforehand (Radford & Joseph, 2020).

##### Theory-driven development

When starting to develop a classification scheme, we recommend inspecting the relevant literature for preexisting classes, which is what we call “theory-driven development”. Theory-driven classes might be inspired by actual classifications (e.g., Ekman’s six basic emotions; Ekman, 1992) or derived by empirical research around the construct of interest (e.g., emotion research investigating awe, thus suggesting an emotion class “awe”; Keltner & Haidt, 2003). The Database of Variables for Content Analysis (DOCA), which collects prior conceptualizations of variables from political and communication research and which aims for comparability and standardization, can be a first starting point (Oehmer-Pedrazzi, Kessler, Humprecht, Sommer, & Castro, 2023a; Oehmer-Pedrazzi, Kessler, Humprecht, Sommer, & Castro, 2023b). If suitable classifications exist, it is essential to test their applicability on a sample from the current data. Preexisting classes might have been developed on different samples, thus covering different expressions of the construct of interest. Furthermore, competing models might exist, and testing the classes on the data can clarify which theoretical approach is more true to the data. Even when the classes are a good match, it is advisable to specify how they can be recognized in the data at hand.

##### Working example

In our working example, one of our goals was to identify the presence of in- and outgroup thinking, that is, whether and how people address their own versus other social groups in their tweets (Tajfel, 1970; Tajfel, Billig, Bundy, & Flament, 1971). For in- and outgroup thinking, we opted for a theoretical specification of classes, since we deemed the space of in- and outgroup thinking well mapped. More precisely, a large body of evidence has shown that people generally favor members of their own group (ingroup favoritism), and perceive members of other groups as inferior (outgroup derogation) (Hewstone, Rubin, & Willis, 2002). Further, there are processes attenuating aforementioned biases, for example, when common group membership is made salient (decategorization) (Gaertner & Dovidio, 2000). Corresponding with previous work on in- and outgroup thinking, we adopted a two-step procedure. First, we classified whether a tweet (our unit of annotation) addressed a writer’s in- or outgroup. Second, we identified the intention of the writer with respect to group identity (i.e., ingroup favoritism, outgroup derogation, or attenuating biases). For the identification of in- and outgroups, we started off with two categories, namely “in” (addressing the ingroup) and “out” (addressing the outgroup). We tested classes “in” and “out” on a sample of n=100 tweets, which led us to conclude that a single tweet can also address both in- and outgroup, or neither. Furthermore, from a reader’s perspective, it can be unclear which group the writer identifies with. This led us to add three additional classes, namely “both” (addressing in- and outgroup simultaneously), “neutral” (addressing neither in- or outgroup), and “unintelligible” (unclear group membership). Next, we aimed to identify the writer’s intention in a tweet regarding group identity. When testing our predefined classes on a sample of n=100 tweets, we found that each tweet can largely be sorted into one of the categories, leading us to keep the proposed classes as is.

##### Data-driven development

Sometimes, preexisting classifications or rich empirical research might not exist for the construct of interest. Because naturally occurring text has unique characteristics, it can be difficult to anticipate which classes might apply. Hence, it is advisable to first study how phenomena occur in the data at hand in a process known as “initial or open coding” (Arthur & Clark, 2021; Glaser & Strauss, 2010; Saldaña, 2013). Nelson, Burk, Knudsen, and McCall (2021) stressed that this flexibility in analysis is perhaps most needed for current, multifaceted social issues, for which stable conceptual frameworks are still unavailable, such as counter speech against online hate.

Techniques from Grounded Theory (Glaser & Strauss, 2010) can be especially helpful for creating classification schemes when adapted to the machine learning context. In “open coding,” the researcher would read the text by iteratively circling between its parts and the whole (the “hermeneutic circle”) and making notes about existing classes and their relationships. The analysis of the text is performed with the fundamental question in mind: “What is going on?” (Franzese & Seigler, 2020). How do people act when they do X? What do they say? What makes us recognize instances of X? Theorizing about the hierarchy and relationship of emerging categories can further lead to hypotheses that can later be tested on the larger data set. For an in-depth discussion of discourse analysis and situational analysis as forms of Grounded Theory see Clarke (2005) and Clarke, Friese, and Washburn (2015).

Recently, other approaches for the data-driven definition of construct categories have emerged that leverage unsupervised machine learning. Compared to supervised machine learning, unsupervised machine learning does not assign predefined labels to the data but instead applies pattern recognition to define classes bottom-up.

One of those approaches is Computational Grounded Theory (Carlsen & Ralund, 2022; Nelson, 2020). It is a methodological framework building on classical Grounded Theory (Glaser & Strauss, 2010) by using natural language processing to make qualitative data analysis more structured, scalable, and replicable. The framework comprises three steps: pattern detection, pattern refinement, and pattern confirmation. In the pattern detection step, researchers apply unsupervised machine learning such as topic modeling (e.g., Latent Dirichlet Allocation; Blei, Ng, & Jordan, 2003) to extract topics at scale and inspire pattern refinement. In step two, computationally extracted patterns are refined through guided deep reading. Finally, in the pattern confirmation step, researchers can apply supervised machine learning, such as text classification, to analyze the entire corpus quantitatively.

Another line of work is the Construct Mining Pipeline (Herderich, Freudenthaler, & Garcia, 2024), which interleaves psychological and computational techniques to identify components of psychological constructs from semi-structured text data. The core of the pipeline consists of collecting short descriptions of (still unknown) components of the construct of interest, which are then embedded with a transformer model into a high-dimensional semantic space, where they can be clustered to infer psychologically relevant construct classes.

For an in-depth introduction to the approaches, we refer to the respective literature (Clarke, 2005; Clarke et al., 2015; Glaser & Strauss, 2010; Herderich et al., 2024; Nelson, 2020).

##### Working example

In our working example, another goal was to measure argumentation strategies that users applied for counter speech. While counter speech strategies have been studied before, the context of the studies was sufficiently different from ours (e.g., experimental or based on the small-scale analysis of selected social media posts; Friess et al., 2021; Benesch et al., 2016; Hangartner et al., 2021). We therefore pursued a data-driven evaluation of argumentation strategies in online political discussions following a Grounded Theory approach.

We drew a random sample of n=1000 tweets from our data sets, balanced across years and the extremity of speech (a variable roughly reflecting users’ political orientation). First, the researcher in charge analyzed the sample with deep reading to accomplish a first draft of the classification scheme and labeled all tweets with respect to argumentation strategy. Afterward, we asked two additional annotators to apply said annotation scheme independently to the same initial sample. In multiple annotator meetings, annotators discussed and resolved disagreements, or merged, discarded, and renamed classes as they saw fit to increase interrater agreement (see also section “Calculating interrater reliability”). Once the classification scheme was final, the two additional annotators relabeled the initial sample independently, forming the basis for our labeling procedure as described in the section “Focus unbalanced training data: iterative annotation.”

### Composing the annotation team

#### Purpose and challenges

When the classification scheme is fixed, a team of annotators usually labels a subsample of the data, which is then used to train the classification algorithm. Here, the best procedure depends on the problem at hand. Annotation teams can take many shapes and sizes, and there is no one-size-fits-all solution. When composing a team, three factors should be considered in general: The type (or expertise) of annotators, the bias that specific annotators introduce to the labels, and the number of annotators (and labels). Since best practices depend heavily on the project, we discuss challenges and solutions for each of these factors in the following.

#### Best practices

##### Type of annotators

Multiple studies have argued that the quality of crowd-sourced annotations reaches the quality levels of expert annotators (Aroyo & Welty, 2015; Snow, O’Connor, Jurafsky, & Ng, 2008), at least if done right (Mitra, Hutto, & Gilbert, 2015; Sabou, Bontcheva, Derczynski, & Scharl, 2014). However, the truth might be more nuanced. Whether expert or crowd-sourced annotations are better may depend on the extent to which the phenomenon can be identified on the manifest or the latent level (Berg, 2009). For example, when annotating medical text, the cause of a disease might be expressed in clear linguistic patterns and might therefore be identifiable by non-experts (e.g., “the erythema nodosum developed *because of* a streptococcal infection”). On the other hand, diagnoses that must be inferred from a set of symptoms might be identified only by experts.

Similarly, studies evaluating the annotations of crowd-workers typically focus on simple linguistic tasks, such as word similarity (Snow et al., 2008). In other, more holistic tasks, such as stance detection with respect to legal debates or framing analyses with a large number of classes, expert annotators show considerably higher levels of interrater agreement (although this is not the only measure of annotation quality, as argued in the section “Calculating interrater reliability”; Gilardi, Alizadeh, & Kubli, 2023).

Furthermore, individual non-expert annotators typically produce labels of lower quality and only when aggregated achieve quality levels of expert annotators (Snow et al., 2008). It is possible that only successful attempts of crowd-sourcing are reported, while unsuccessful attempts are not, biasing the field’s confidence in crowd-sourced annotation. Finally, if expert annotators are involved in data annotation, they can later help to substantiate the statistical analysis by tying the quantitative analysis to the qualitative interpretation of the analyzed classes.

Another choice concerns the use of generative pretrained transformers (GPTs) as annotators. GPTs (e.g., OpenAI’s ChatGPT) are LLMs that rely on the question-and-answer principle, where users “prompt” the model with a natural-language request and the model outputs a natural-language answer. Recent advances in the training of foundation models, especially in ChatGPT (GPT-5), have made this option viable. Although GPTs have the same underlying architecture as traditional transformer models (e.g., BERT), at a much larger scale, they are qualitatively different. Several studies evaluated the quality of GPT-annotations (models 3.5 or 4), and found that across different tasks (such as political stance or sentiment detection) and languages, GPT outperformed simpler models, including customized BERT models, as well as crowd-workers and expert annotators (Gilardi et al., 2023; Heseltine & Clemm Von Hohenberg, 2024; Törnberg, 2025). On the other hand, GPT’s accuracy was related to the complexity of the task (Gilardi et al., 2023; Heseltine & Clemm Von Hohenberg, 2024), indicating that GPTs likely perform better on simpler annotation tasks. Still, the increase in LLM capabilities introduces another challenge: Even with preventive measures in place and given LLMs’ easy access, researchers cannot be fully sure whether annotations obtained via crowd-working platforms are manual or automated.

Other than using GPTs as annotators, they can be engaged as classification models directly. That means GPTs are prompted to fulfill the classification task, circumventing the data annotation step. The instructions that the model receives to complete the annotation task should be well documented in a “promptbook” (Stuhler, Ton, & Ollion, 2025), which next to the specific prompts, can also include steps taken to improve the prompts and prompting techniques employed. In the present guidelines, we discuss mixed evidence for the suitability of GPTs as classifiers and include experiments on using GPT-4 compared to training a specialized classification model based on (human) annotated data (see the section “LLMs and traditional transformers as classifiers”).

##### Working example

In our study on counter speech, we first attempted to collect annotations via crowd-sourcing, but failed to establish even the most basic levels of interrater reliability (i.e., Krippendorff’s alpha was .2 or lower) and therefore decided to switch to expert annotators. Our annotation team consisted of four psychologists with a broad interest in and knowledge about local politics. The team later helped us to establish hypotheses about the mechanisms behind the observed effects. For example, the expression of positive emotions failed to increase discourse quality likely because positive emotions would often be used to portray the ingroup as superior while rallying against the outgroup. As such, access to expert annotators over the entire course of a project is an invaluable resource for the interpretation of findings.

##### Annotator bias

Especially for annotation tasks related to strongly contested issues, such as hate speech, researchers should consider biases introduced by annotators’ characteristics. For example, studies have shown that conservative annotators perceive anti-black speech as less toxic and African American English as more toxic (Sap et al., 2022). Increased sexist beliefs relate to a less accurate detection of misogyny, and the structure of crowd-working platforms can amplify these biases (Hettiachichi et al., 2023). As such, researchers should consider which biases are relevant to their research questions and balance respective personal context variables (e.g., socio-demographics, political orientation, or ethnicity) within the annotation team. Personal context variables of the annotators are ideally reported with the release of the data sets (Gebru et al., 2021; Geiger et al., 2020, 2021). If investigators annotate the data directly, they should consider issuing a “positionality statement”, which is already a common and essential part of qualitative social science research.

##### Working example

Our annotators were all female in their 20s, all identifying as politically left or center to left. We tried to mitigate potential biases through annotation instructions; however, ideally, our team should have been more diverse.

##### Number of annotators

The question about the number of annotators goes hand in hand with the question about the number of labels desired per data point. Based on our experience, we do not recommend obtaining only one label per sample, at least not for the whole training data set, since disambiguation of context-dependent or subjective constructs will always be necessary for successful classifier training (as illustrated in “Focus model performance: training on confident examples”). Still, there is no definite answer on how many labels per data point to obtain. Similar to the choice of the annotation team and the overall size of the training data (see the section “How much annotated data is enough?”), it depends on the ambiguity of the measured concept, the chosen label aggregation strategy, and ultimately on the monetary resources for labeling.

##### Working example

Our annotation team consisted of four annotators, which was a trade-off between the following factors: Availability, monetary resources, the anticipated time to annotate the entire training data given four annotators, and the fact that at least three annotators are necessary to compute majority labels, or, in other words, to disambiguate diverging labels.

### Sampling the ideal training data

#### Purpose and challenges

One key question after both the classification scheme and the annotation team are defined is which subset of the entire data, with often millions of texts, should be annotated. Here, we discuss four subjects with respect to the training data: the size of the training data, interrater reliability, as well as sampling strategies for both the training set (used to train the machine learning classifier) and the test set (used to evaluate the final classification model).

One of the biggest challenges when working with naturally occurring text (i.e., human text primarily generated for purposes other than research) is the near-complete lack of researcher influence on the data-generating process. While questionnaires, surveys, and interviews are tailored to assess a targeted phenomenon and rule out potentially confounding factors, naturally occurring text is noisy, biased (Barberá & Rivero, 2015) and might only contain small numbers of relevant instances. Moreover, dynamics on social media are the result of interactions between humans and algorithms (McLoughlin & Brady, 2023) further obfuscating what we are trying to observe in digital trace data specifically. This raises the question of how much data needs to be annotated to cover all facets of the constructs that we are interested in (see the section “How much annotated data is enough?”).

Another challenge resulting from potentially biased, naturally occurring text is to establish annotation quality in the form of sufficient interrater agreement, also known as interrater reliability (IRR). There is an ongoing debate on IRR indices and their interpretation in the literature. Studies show that IRR can vary considerably depending on the chosen indicator (e.g., .10 to .92 for interval-scaled data; ten Hove, Jorgensen, & van der Ark, 2018). In addition, common standards (Landis & Koch, 1977) were once developed for a specific indicator, Cohen’s kappa, and were uncritically transferred to other indicators later on. In the section “Calculating interrater reliability”, we review best practices around IRR indices.

Furthermore, how the training data is selected can have a substantial impact on the classifier’s performance and the validity of the results. Machine learning models can be less accurate when the meaning of concepts drifts over time (Koh et al., 2021). In addition, machine learning algorithms can be prone to learning contexts instead of constructs, which becomes a problem if contexts in the training data are different from the data on which inference is performed. In the section “Sampling strategies for training data”, we discuss solutions for both concept and context drifts.

When training machine learning classifiers, it is crucial to evaluate their performance against a gold standard data set established by human annotation. The best practice is to hold back a portion of the annotated data that is not used for training, but to evaluate the performance of the classifier after training is finished. Given that sociological and psychological concepts are judged differently in text by humans, the question of how to compose a gold standard test set for labels on which annotators disagree arises (see the section “Creating a held-out test set”).

Finally, over- or underrepresentation in the training data is an issue for the classes the machine learning classifier is supposed to identify. In an extreme example where class A is only present in 5% of the training data while class B makes up 95% of the training data, the classifier could simply ignore the underrepresented class, since errors due to misclassification of the very rare examples of A do not contribute much to the overall error. Since the class of an observation is not known beforehand, simply oversampling so-called minority classes when composing the training data set is not an option. The problem of class imbalance can, however, be addressed with what we call “Iterative Annotation”, a new strategy we discuss in the section “Focus unbalanced training data: iterative annotation”.

#### Best practices

##### How much annotated data is enough?

Similar to the question of how many and which annotators are needed, there is no universal answer to the problem of selecting a sufficiently large data set for annotation. In general, machine learning needs large amounts of data to learn strategies to solve a problem reliably, since – unlike humans – it relies solely on statistical learning for inference, and the data requirements can increase depending on the problem. Several factors increase the need for training data, two of which are a large number of classes and the ambiguity of the measured concept (i.e., its intersubjectivity and context-dependency).

The question of sample sizes for annotated data is a problem that has previously been given little attention (Chang et al., 2023). A possibility to empirically estimate the sample size is to increase training data only if classifier accuracy is still insufficient (Chang et al., 2023), that means classifier accuracy is monitored during training and the amount of training data is only increased if classifier accuracy is still insufficient. To judge model quality, human performance (e.g., IRR) has been argued to be an upper bound for model accuracy (Kjell, Sikström, Kjell, & Schwartz, 2022; Novak et al., 2022).

Importantly, “bigger is better” is not always true for data in machine learning (Longpre et al., 2024) and it is a common myth that the correctness of inferred relationships improves the larger the size of the training data set (Németh, Sik, & Máté, 2020). The quality of the training data influences the model’s performance, but creating high-quality data is often laborious. The model learns best when given high-quality data with informative samples to distinguish edge cases; the remainder of this section is dedicated to strategies for obtaining such data.

##### Working example

At the beginning of our study, we aimed for roughly 10,000 annotated examples. We operated under the assumption that, given the IRR we deemed sufficient, it would be sufficient to collect one expert label per sample. However, as demonstrated under the section “Focus model performance: training on confident examples”, we ended up using only a fraction of the data with two or more labels for model training. This demonstrates nicely how “bigger is better” does not necessarily hold, specifically for ambiguous classification problems as can be found in the social sciences.

##### Calculating interrater reliability

Calculating interrater agreement is a means to secure data quality – if annotators tend to agree, the construct of interest is measured reliably. Most modern IRR coefficients, such as the widely used Krippendorff’s alpha, were designed to improve upon percentage agreement by adjusting for chance agreement. However, these indicators assume that raters intentionally try to rate as random as possible, where a more realistic assumption is that random ratings are involuntary and task-dependent (Zhao, Feng, Ao, & Liu, 2022).

In an empirical study that allowed to derive the true expected IRR, Zhao et al. (2022) showed that among seven popular indices, percentage agreement surprisingly predicted true IRR most accurately (r=.84), while Krippendorff’s alpha and similar indices adjusting for chance agreement did not (r=.31). Krippendorff’s alpha, Conger’s kappa, and Fleiss’ kappa indicated skewness above anything else (Quarfoot & Levine, 2016; Zhao et al., 2022). The empirical study further introduced a new characteristic to judge the quality of IRR indices, namely task difficulty, which had the strongest influence on reliability, even before factors like the number of categories and skewness. However, until today, no index considers task difficulty as an influencing factor.

Other studies have noted that maximizing IRR should not be the sole goal when constructing a labeled data set, since other important quality characteristics such as context dependency and annotator diversity might actually drive down agreement (Arhin, Baldini, Wei, Ramamurthy, & Singh, 2021). Alternatives to common reliability indices are Gwet’s AC1 and AC2 (Gwet, 2014) and the Brennan–Prediger coefficient (Brennan & Prediger, 1981), which are less known, but also less prone to skewness in the data (Quarfoot & Levine, 2016); as well as simply estimating percentage agreement and correcting for its average overestimation (minus 15 percentage points as proposed in Zhao et al., 2022).

**Table 2:** Different interrater agreement scores on our held-out test set

	strategy	group	goal	hate
Krippendorff’s alpha	.45	.42	.45	.50
Fleiss’ kappa	.45	.42	.45	.50
Conger’s kappa	.45	.43	.45	.50
Brennan–Prediger	.48	.51	.55	.64
Gwet’s AC1	.48	.52	.57	.67
Percentage agreement	.52	.59	.61	.73
Percentage agreement -15%	.37	.44	.46	.58

*Note.* Measurements between four annotators with n=997 data points. Calculations were performed in Python (version 3.10.14) using the package irrCAC (version 0.4.1). The four columns correspond to the four concepts we annotated in our data from the working example: strategy = argumentation strategy, group/goal = in- and outgroup content, hate = hate speech

##### Working example

In our study, we reached Krippendorff’s alphas between .42 and .50 on the test set. This is low with respect to traditional standards (Landis & Koch, 1977), but not necessarily an indicator for low data quality. There are several factors to contextualize IRR values in our working example and other studies: (1) We took measures to improve IRR, including switching from crowd-workers to expert annotators, and revising the classification scheme in several rounds in annotator conferences. (2) We introduce a new training strategy for machine learning classifiers (“Training on Confident Examples”) that prioritizes data points with high agreement. (3) We introduce a new analysis strategy that accounts for classifier inaccuracies in the statistical analysis (“Classifier Based Bootstrapping”) making the conclusions robust to classification errors.

Table 2, which collects different agreement coefficients for our data, shows that values can differ by up to 20 percentage points depending on the chosen indicator. Krippendorff’s alpha, Fleiss’ kappa, and Conger’s kappa, all prone to skewness, provide similar estimates of IRR, while the Brennan–Prediger coefficient and Gwet’s AC1, consistently estimate IRR to be higher.

##### Sampling strategies for training data

If data is not distributed uniformly over time, the machine learning classifier will systematically perform worse for data from time periods that were underrepresented in the training data. To counteract this effect, data can be subdivided into a number of bins representing time periods and the training data set can be composed by sampling the same number of examples from each bin.

A similar argument applies to other known attributes of observations that are of interest for the analysis or that could influence constructs of interest, such as demographic characteristics or region. Whenever training data is not distributed uniformly across a potentially confounding factor, it is advisable to subdivide the data based on this variable into bins and sample equal numbers of examples from those bins to be annotated.

##### Working example

For our example case, we had data from the years 2015 to 2018, with over 40% of the data coming from the year 2018 alone. In our training data set, however, each of the years contributes 25% of training examples.

In our case, tweets from accounts similar to Reconquista Germanica (RG, hate speech group) were most prevalent, followed by tweets from neutral accounts, while tweets from accounts similar to Reconquista Internet (RI, counter speech group) were least prevalent. However, our research question was connected to counter speech strategies, and we expected the most relevant tweets to come from accounts similar to RI. To ensure that our machine learning classifier had good performance on tweets from all account types, we oversampled accounts that were similar to RI as well as neutral accounts for our training data set.

**Fig. 2:**
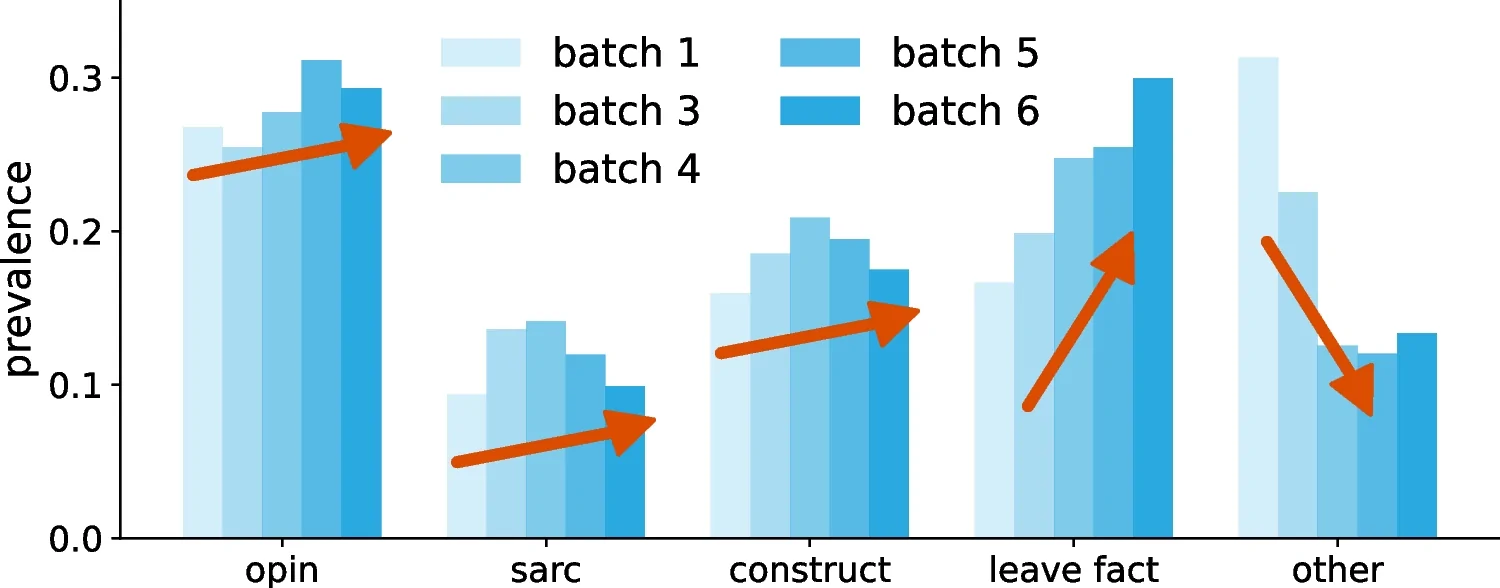
Prevalence of argumentation strategies after six iterations of data labeling. *Note.* Prevalence refers to the percentage of tweets belonging to a given argumentation strategy in a given batch of annotated data. The bar on the left (*light blue*) refers to the thousand initially annotated tweets (batch 1). With “Iterative Annotation” minority classes (sarcasm (sarc), constructive comments (construct), and leaving factual discussion (leave fact)) increase, while majority classes (opinion (opin), and other) decrease over consecutive batches of annotation

##### Creating a held-out test set

After the training of the machine learning classifiers is finished, it is best practice to evaluate the performance of the classifiers against a held-out test set, that is, data that the classifier did not see during training. This is also known as the “ground truth”. While some classification problems (like classifying images of dogs versus cats) have a clear and objective ground truth, most problems in the social sciences are more ambiguous. Researchers are therefore discussing whether ground truth exists and are proposing procedures where multiple interpretations of the same data point can co-exist and even be informative (Aroyo & Welty, 2015; Cabitza, Campagner, & Basile, 2023).

Cabitza et al. (2023) review strategies to resolve disagreement among multiple raters, an approach called data perspectivism. Perspectivism can further be distinguished into “weak perspectivism” and “strong perspectivism”. In weak perspectivism, disagreement is resolved by consolidating multiple labels into a single label, whereas in strong perspectivism, classifier training is used to leverage multiple disagreeing labels for the same instance.

For example, strategies for weak perspectivism include collecting confidence scores of annotators’ judgments along their labels and performing label aggregation with a weighted majority vote. Another possibility is to reduce the instances of the test set to include only the cases where a majority of the annotators agree (see also “Working example”).

Strategies for strong perspectivism include converting a classification task into a regression task by associating each sample with a confidence score and predicting the presence or absence of each label separately. For example, if a sample received three labels A, A and B, the sample would receive a score of 0.66 for A and 0.33 for B, and we would predict the scores for A and B with two classifiers separately. Another possibility is to duplicate the samples and associate each duplicate with one label from one annotator. That said, if three annotators annotate a sample, there would be three duplicates of the sample with a single label. A third possibility is to leverage ensemble learning by training one classification model per annotator or a model that jointly predicts each annotator’s labels and the majority vote, and aggregate the model predictions. Along the same lines, the training data can be bootstrapped to train multiple classifiers, and the classifier predictions can then be aggregated.

Cabitza et al. (2023) further note that the development of techniques to exploit multiple labels per sample is still ongoing.

##### Working example

We drew a sample of 1000 tweets to create a held-out test set. This sample was balanced across time and account similarity to hate and counter speech groups, and was annotated by all annotators. We decided to only include tweets for which at least three out of four annotators agreed on the label (“unanimous” and “strong majority”), which aligns with interpretation through consensus as in Németh et al. (2020) when interrater agreement is low. As such, we obtained a final held-out test set of 594 tweets, out of which 187 were labeled as “opinion,” 164 as “leaving factual discussion,” 144 as “other,” 62 as “constructive” and 37 as “sarcasm,” demonstrating the class imbalance in our data as well.

#### Focus unbalanced training data: iterative annotation

Other than an imbalance in confounding variables (see the section “Sampling strategies for training data”), imbalance of the classes that the machine learning model is supposed to predict in the training data can be a problem since minority classes are simply ignored by the classifier during training. “Iterative Annotation” is a strategy that can be employed to address class imbalance in the training data. Inspired by Metzler, Baginski, Niederkrotenthaler, and Garcia (2022), the aim of this approach is to bias the sample selection process such that a sufficient number of examples from each class is included in the final training data set. Other sampling strategies to counteract class imbalance exist (e.g., random oversampling or synthetic minority oversampling; Stoll, 2020), however, these are only applied post hoc after the annotations have already been collected and do not add new information to the data.

For “Iterative Annotation”, in our study, we first selected a thousand random samples balanced with respect to their creation date and the similarity to known hate and counter speech groups (confounding variables). We annotated these samples following our classification scheme, which resulted in a very unbalanced prevalence of the different argumentation strategies, as illustrated in Fig. 2 (light blue bar on the left).

We trained a preliminary classifier on the first thousand annotated examples to predict the argumentation strategies. We could not successfully train the more sophisticated transformer model that we intended to use for the final classification to recognize several of the minority classes (see also the section “Selecting an adequate base model”). Therefore, we initially trained a simpler model called a support vector machine (Cristianini & Ricci, 2008; Pedregosa et al., 2011). Using a simpler model for “Iterative Annotation” before changing to the final model can generally reduce the time spent training the preliminary model.

We used the preliminary classifier to infer class probabilities in the remaining unlabeled corpus and assigned each tweet the class with the highest probability as a preliminary label. We then drew another batch of samples only selecting tweets classified as minority classes (here: “constructive comments”, “sarcasm”, and “leaving factual discussion”). Given the insufficient initial performance of our preliminary classifier with F1 scores ranging between 0.2 and 0.5 (see Appendix C for an overview of performance metrics), this increased the prevalence of tweets labeled as minority classes by the human annotators while still including plenty of tweets from majority classes. The new batch of samples was again annotated by human annotators and, together with the tweets in the first batch, used to train a new version of the preliminary classifier to improve its performance gradually.

**Fig. 3:**
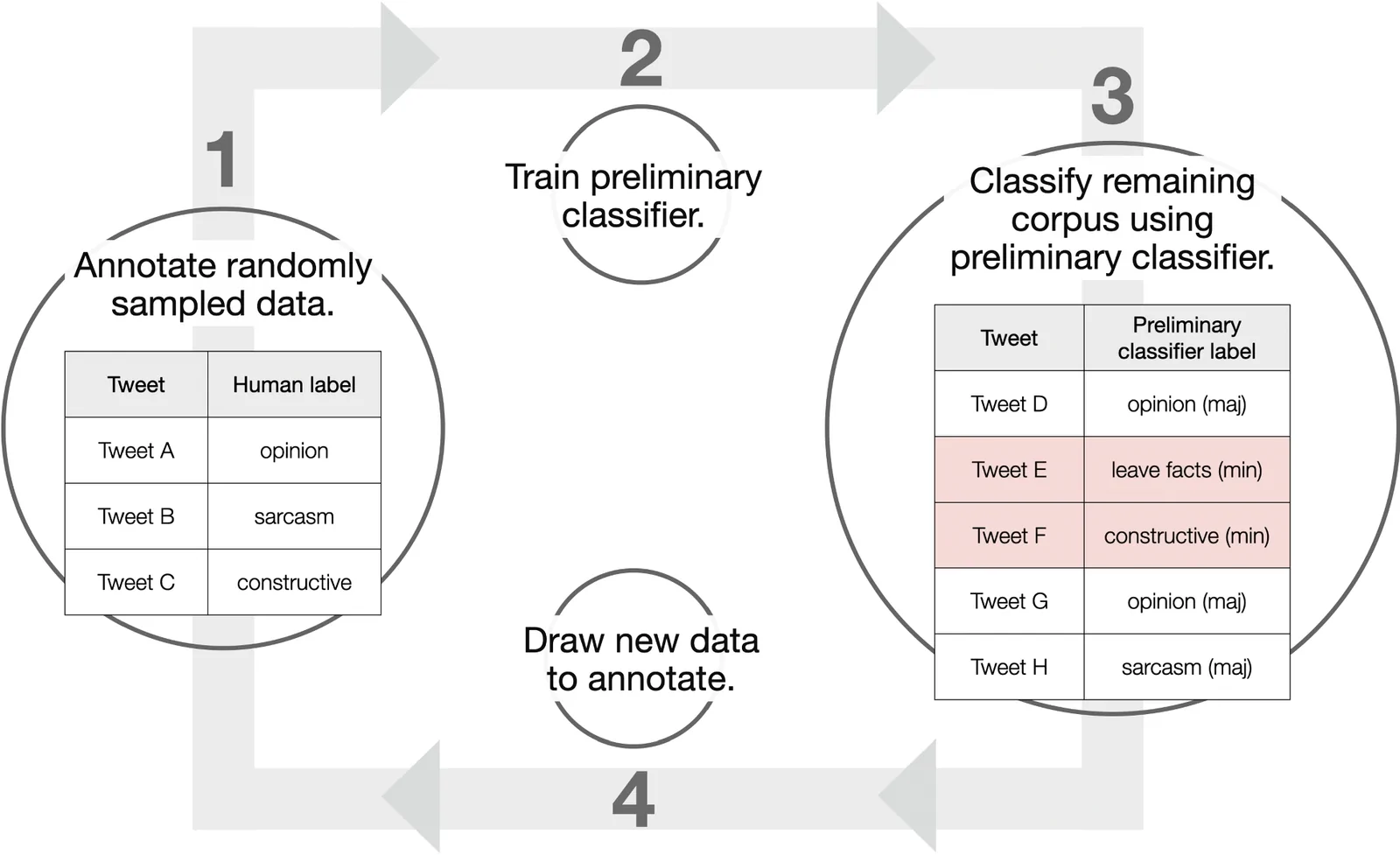
Iterative annotation for unbalanced training data. *Note.* “Iterative Annotation” is a strategy to counteract unbalanced training data and therefore to enhance classifier performance. Randomly sampled data should additionally be balanced over confounding variables. Preliminary classifiers can be simpler models (e.g., a support vector machine), and the target model (e.g., transformer model) may only be implemented after several iterations. When drawing new data to annotate, only samples labeled as minority classes by the preliminary classifier are drawn. The process is repeated over multiple rounds. min = minority class; maj = majority class

We repeated the sampling, annotation, and training process multiple times. After several iterations, we switched the preliminary classifier to our final model architecture. The effect of “Iterative Annotation” is illustrated in Fig. 2. As expected, minority classes such as “leaving factual discussion” increased sharply in the training data, while other (irrelevant) classes such as “other” decreased. Figure 3 illustrates the process of “Iterative Annotation”.

## Model training

### Selecting an adequate base model

#### Purpose and challenges

Machine classification of text can be achieved in many different ways, from traditional machine learning architectures such as logistic regression, decision trees, or support vector machines (James, Witten, Hastie, Tibshirani, & Taylor, 2023a; Pedregosa et al., 2011) to sophisticated transformer models and LLMs. At the beginning of training a classification model, it is best practice to compare several models against each other (with respect to different performance indicators, see Appendix C) to make an informed decision about which architecture fits the problem at hand best.

When it comes to natural language understanding tasks, machine learning models employing a transformer architecture have become the standard in the field. Since the publication of the first transformer architecture (Vaswani et al., 2017), a large number of open-source models have become available. Models pre-trained to understand a particular language can be fine-tuned on a smaller data sets to learn a specific classification task. Hugging Face is the platform through which many of those models are freely accessible.

With the introduction of OpenAI’s GPT-3.5, LLMs as general-purpose models have become feasible for tasks such as text classification and are now a viable alternative to smaller transformer models trained for specific tasks. LLMs are capable of performing many natural language processing tasks with or without further training (Rathje et al., 2023). However, performance of LLMs on classification tasks is sometimes below that of fine-tuned smaller language models tailored for specific tasks (Aroyehun et al., 2023; Kocoń et al., 2023; Ziems et al., 2024). Additionally, LLMs as classifiers can threaten the reproducibility and validity of scientific findings. In the following section, we will discuss advantages and disadvantages of LLMs as classification models with a special focus on social science concepts backed up by a simulation of an LLM classification with our working example.

#### Best practices

##### LLMs and traditional transformers as classifiers

The application of LLMs for measuring constructs requires a consideration of various important factors. One critical concern is the sensitivity of LLMs to the phrasing and structure of prompts, introducing unpredictability in outputs and posing challenges for researchers aiming to obtain consistent and reliable results (Demszky et al., 2023). Although prompting possibilities are endless, “promptbooks” offer a means to document the prompts alongside the prompt engineering process (Stuhler et al., 2025). Another crucial factor involves the moderation (sanitization) of prompts, input, and output from LLMs accessed through APIs, as service providers often provide limited details that can impact the validity of projects that rely on LLMs. Limited or non-existent access to the confidence of model outputs may hinder researchers’ ability to assess the reliability and uncertainty of LLM predictions. Finally, classification with LLMs is limited by a model’s context window, that is, the number of tokens (or words) the model can process simultaneously. This puts a limit on the detail with which the prompt and thus the classification scheme can be formulated.

Moreover, the issue of performance variation across languages is a challenge, with research demonstrating higher performance levels in English compared to other languages (Lai et al., 2023; Zhang, Aljunied, Gao, Chia, & Bing, 2023). This can be attributed to several factors, including the availability and quality of training data in different languages, linguistic nuances, and cultural context. Dependency on external APIs for LLM access limits researchers’ ability to account for and control model updates, versioning, availability, and potential service interruptions. This can affect the feasibility of achieving long-term research objectives and their reproducibility. Financial considerations also come into play, as the costs associated with accessing LLMs through APIs or running one locally can be prohibitively expensive, particularly for researchers with limited financial resources or when analyzing large amounts of data.

Regardless of these concerns, recent literature (e.g., Kumarage, Bhattacharjee, & Garland, 2024; Nirmal, Bhattacharjee, Sheth, & Liu, 2024; Bhattacharjee & Liu, 2023; Bhattacharjee, Moraffah, Garland, & Liu, 2024; Matter, Schirmer, Grinberg, & Pfeffer, 2024) has increasingly demonstrated the effectiveness of LLMs as reliable tools for annotating or classifying within a question-answer framework (where a user prompts an LLM with a question and receives an answer). At the time of our study, performing classification tasks with LLMs was still not an option. However, to explore this potential within our specific area of interest, we conducted three experiments using OpenAI’s GPT-4. These included two tests where we provided GPT-4 with a codebook: a Zero-Shot experiment, which relied solely on definitions without examples; and a Few-Shot experiment, where in addition to the codebook, one example from each category was given. In our experiments, we classified tweets along their mentions of ingroups and/or outgroups (Tajfel, 1970; Tajfel et al., 1971) with the classes “exclusionary about outgroup,” “inclusionary about in- or both groups,” and “other.” Third, we carried out a Few-Shot experiment without providing the codebook, only showing GPT-4 one random example from each category with a corresponding label. For each experiment, we selected tweets for which a majority of human annotators concurred, treating the majority-agreed label as the truth. This experiment was conducted 100 times. The exact prompts and labels provided to GPT-4 under each experimental setting can be found in Appendix A.

According to our results (see Table 3), examples provided without the codebook’s instructions yielded better performance, suggesting that the complexity of the instructions might confuse GPT-4. This aligns with findings in existing research, which indicates that straightforward labeling instructions often surpass more intricate ones. On the other hand, this could be interpreted as GPT-4’s failure to base its judgment on meaning construction rather than linguistic patterns alone (Németh et al., 2020). For a cost estimation of using GPT-4 as an annotator or classifier with respect to our example, we refer to Appendix B. For more discussion, Kumarage et al. (2024) and Tan et al. (2024) offer comprehensive reviews of the application of LLMs as classifiers.

As LLMs as classifiers were not yet suitable at the start of our project, in our working example, we ended up choosing “twitter-xlm-roberta-base” (Barbieri, Anke, & Camacho-Collados, 2021) as our base model. This decision was based on the language(s) and text type the model was pre-trained on, the model’s parameter count (which translates into computing power requirements), and the model’s pre-fine-tuning prediction accuracy variability.

**Table 3:** Performance of GPT-4 under three different conditions

Experiment type	macro-F1
Human coders/classifier	0.488
Zero-shot w/CB	0.502
Few-shot w/o CB	**0.626**
Few-shot w/CB	0.429

*Note.* We used GPT-4 to annotate a small sample of n=100 tweets using the classes “inclusionary about in/both groups,” “exclusionary about outgroup,” and “other” for three different experimental setups: Zero-shot with codebook and few-shot with and without codebook. These tweets were chosen from the held-out test set in which the majority of human annotators reached consensus on a label. The highest performing experiment is presented in bold. Here, few-shot means that a random example from each class was provided to the LLM prior to classification. w/CB: with codebook, w/o CB: without codebook

### Evaluating model performance

#### Purpose and challenges

The performance of classification models is evaluated during and after training to guide model development and to judge the performance of the final classifier against human annotators. Multiple indicators exist that come with different advantages and disadvantages. In the following, we provide a brief introduction to the most common indicators for evaluating classification models during and after training.

**Fig. 4:**
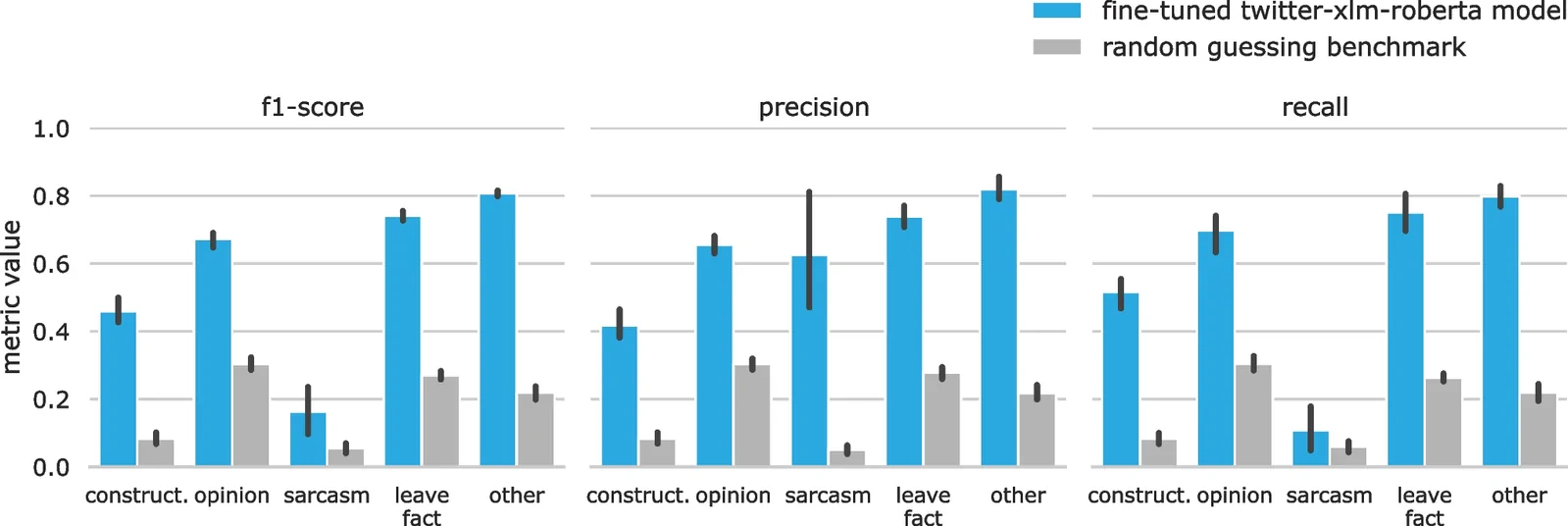
Macro-averaged F1 score, precision, and recall of the final machine learning classifier for argumentation strategy. *Note.* *Error bars* indicate 95% confidence intervals calculated via fivefold cross-validation. *Grey bars* indicate the performance of a frequency-based random guessing benchmark. Performance is depicted for each class of argumentation strategies separately: construct = constructive comments, opinion, sarcasm, leave fact = leaving factual discussion, other = none of the above

#### Best practices

##### Final performance evaluation

When comparing the output of a machine learning model against a predefined gold standard data set (see the section “Creating a held-out test set”), a variety of indicators exist to choose from. We include a brief introduction to accuracy, F1 score, receiver operating characteristic (ROC) and area under the curve (AUC), as well as the confusion matrix in Appendix C. It makes sense to use the same evaluation metric for model development and final assessment, but the data sets used differ. Depending on the application, other evaluation criteria such as fairness (i.e., classification accuracy between subgroups) can be relevant. There is substantial disagreement on how to quantify fairness, and metrics are less standardized. For an overview of fairness metrics, we refer to Caton and Haas (2024).

##### Working example

In Fig. 4 we show the performance of our classifier for argumentation strategy using F1 score, precision, and recall. Providing all of these metrics allows for an assessment of the classifier on different tasks, e.g., how good it is at not incorrectly identifying a class (precision) and how good it is at identifying all examples of a given class (recall). The F1 score is the harmonic mean of precision and recall and one of the most commonly used indicators to assess classification performance.

##### Evaluation during model development

During model development, we cannot use the held-out test set for evaluation, because we are interested in the model’s performance on unseen data. During model development, it is therefore customary to set aside a portion of the available training data for use as a validation data sets. Splitting the data in this way multiple times with random assignment of observations to the training and validation portion of the data set (*n*-fold cross-validation) can further be used to assess the variance of model performance (see Fig. 4).

The number of folds depends on the size of the data set and the available computing time. If *n* is chosen to be higher, the model sees a larger number of examples during training and is tested on a smaller test data set. As the model sees more of the available data, this should lead to a lower prediction error (Olsen, 2024). However, since doing *n*-fold cross-validation with a high *n* is computationally expensive, choosing a lower *n* might be necessary. Typical values established in the literature are n=5 and n=10 (James, Witten, Hastie, Tibshirani, & Taylor, 2023b).

##### Working example

In our project, we used fivefold cross-validation. For every split, we assigned 85% of the data to the training data set and 15% of the data to the validation data set. During model training (see also the section “Improving model performance”), the performance on the validation set was both used to decide on model hyperparameters as well as on when to stop model training.

### Improving model performance

#### Purpose and challenges

To give our chosen model (see the section “Selecting an adequate base model”) the capability to act as a classifier, we need to train or “fine-tune” the model further on a data set of examples and class labels. This process will update the model’s weights, including its “head,” the layer that transforms the model output into a class prediction.

In the following, we outline the decisions to be made during this training step. There are different techniques to improve model performance, but which of those works best for a specific application case is hard to tell beforehand. Among the techniques we are discussing are Masked Language Modeling (MLM), hyperparameter tuning, loss functions, and early stopping, as well as a new technique called “Training on Confident Examples” introduced in the section “Focus model performance: training on confident examples”. We illustrate training decisions with our working example, for which we used the Transformers library (Wolf et al., 2019) for Python that builds on the machine learning framework PyTorch (Paszke et al., 2019). Packages for transformers in R exist (Kjell, Giorgi, & Schwartz, 2023), but usually have limited capabilities compared to Python. We further relay our experience on which techniques work best in the section “How good is good enough”.

#### Best practices

##### Masked language modeling

Masked Language Modeling (MLM) is also called domain-adaptive pre-training and has the potential to improve model performance in the target domain (e.g., social media posts; Gururangan et al., 2020). Analogous to the initial pre-training of the model, MLM is a self-supervised task, that is, the model learns from the data alone without providing external labels. To this end, the model first masks a predefined percentage of tokens (which can be roughly thought of as words) to then predict a token that has been masked, given its surrounding context.

##### Working example

We pre-trained the twitter-xlm-roberta-base model with a MLM task on the full corpus of 1,167,853 tweets. We performed MLM with a masking probability of 15%, cycling through all available data 100 times (also called 100 “epochs”), using a randomly selected 20% of the corpus as a validation set. The MLM task took 142 hours to complete on a single NVIDIA Quadro RTX 8000 GPU with 48 GB GDDR6 memory. When assessing classification performance of the pre-trained versus original model, we observed performance gains of 0.03–0.05 in macro-averaged F1 scores.

##### Tuning model hyperparameters

When fine-tuning a transformer model, a number of model hyperparameters can be chosen that influence the training process. Hyperparameters are values the researcher provides to the model to set the boundaries of model training, rather than parameters learned from the data. A short summary of the function of the most important hyperparameters for transformer models can be found in Appendix D, which in our experience were the training batch size, learning rate, warm-up steps, label smoothing factor, and the weight decay.

Even when only tuning five hyperparameters, the space of possible hyperparameter combinations that could be tried is very large, resulting in an infeasible amount of time spent on re-training the model. A common strategy to circumvent this problem is to conduct a random grid search (Bergstra & Bengio, 2012). In a random grid search, a set of values is defined for each of the hyperparameters to be chosen. From this parameter space, random combinations are selected for training the model and comparing performance. This could be followed by a search in a small area of hyperparameter values around the best combination found in the random search.

##### Working example

We opted for a random grid search (Bergstra & Bengio, 2012) of possible hyperparameter combinations in the following parameter space: learning rate: [$1\cdot 10^{-5}$, $5\cdot 10^{-5}$, $1\cdot 10^{-4}$], weight decay: [0.001, 0.0025, 0.005], label smoothing factor: [0.1, 0.2, 0.3], training batch size: [32, 64, 128, 256, 512]. We kept the number of warm-up steps at the model’s default value. Since differences in model performance between different hyperparameter combinations were relatively small (F1 score differences of 0.01–0.03), we did not invest time in further exploring the parameter space and chose the best combination of hyperparameters from the random grid search, which were a learning rate of $5\cdot 10^{-5}$, weight decay of 0.0025, a label smoothing factor of 0.2 and a training batch size of 256.

##### Loss function and early stopping

In order to update the model weights during fine-tuning, the “loss function” of the model is calculated, which quantifies the difference between the model’s current prediction and the known target values of the training data set. The loss function is usually the cross-entropy in a classification task (Cover, 1999). The goal of fine-tuning is to minimize the loss function. This can be achieved using “gradient descent.” The gradient (i.e., the rate of change) of the loss function with respect to the model weights indicates the direction in which model weights should be changed to minimize loss.

When fine-tuning a pre-trained model, the process of prediction, calculation of the loss function, and updating of model weights is done for every batch of training data that the model sees at once. In addition, the available training data is cycled through several times, also called “epochs.” If the loss has not substantially decreased between two (or more) successive epochs, training can be stopped to save time (“early stopping”).

##### Working example

We employed early stopping, halting training if the loss did not decrease, but increased over five consecutive epochs. The model is set to the iteration before the increase in the loss function.

##### How good is good enough?

A question that remains to be answered is when a model’s performance is good enough to achieve the intended task, here, answering a research question. While slight improvements in model performance are always a possibility when adding more training data or finding a more optimal configuration of model hyperparameters, the investment of time and money to do so might not be warranted by the possible gains.

In our experience, there is no hard rule for when a model is good enough. The statistics literature provides rules of thumb (see also our discussion of performance metrics in Appendix C and IRR in the section “Calculating interrater reliability”), but the context of the research question and nature of the intended analysis play an equally important role. If the following statistical analysis allows for a robust treatment of the uncertainties introduced by the machine learning classification, even relatively poor classification performance can be acceptable since it will not result in biased results but merely in enlarged confidence intervals (see the section “Accounting for classifier inaccuracy” including “Focus group comparisons”).

Therefore, here we cannot give any general recommendations on what model performance to aim for. We can, however, relay our experience in what efforts tend to contribute most to model performance.

##### Working example

In our experience, the biggest contribution to how well a model is going to perform in a classification task is the quality of the training data, especially for classifications with a high degree of ambiguity. At the start of the project, we operated under the assumption that more data is always better. When our models failed to achieve acceptable performance levels, we circled back to the training data. Changing our model training strategy from using all available training data, including samples with only a single human label, to using only samples with two or more labels improved macro-averaged F1 scores by 0.15 to 0.2, even though this reduced the number of available training samples from over 14,000 to just over 2000. We discuss our new strategy, termed “Training on Confident Examples” in detail in the next section “Focus model performance: training on confident examples.” The second most effective strategy to increase model performance was to extend model pre-training (section “Masked language modeling”), leading to improvements in macro-averaged F1 scores by 0.03 to 0.05. Tuning model hyperparameters (section “Tuning model hyperparameters”) had an almost negligible impact on classification performance.

**Fig. 5:**
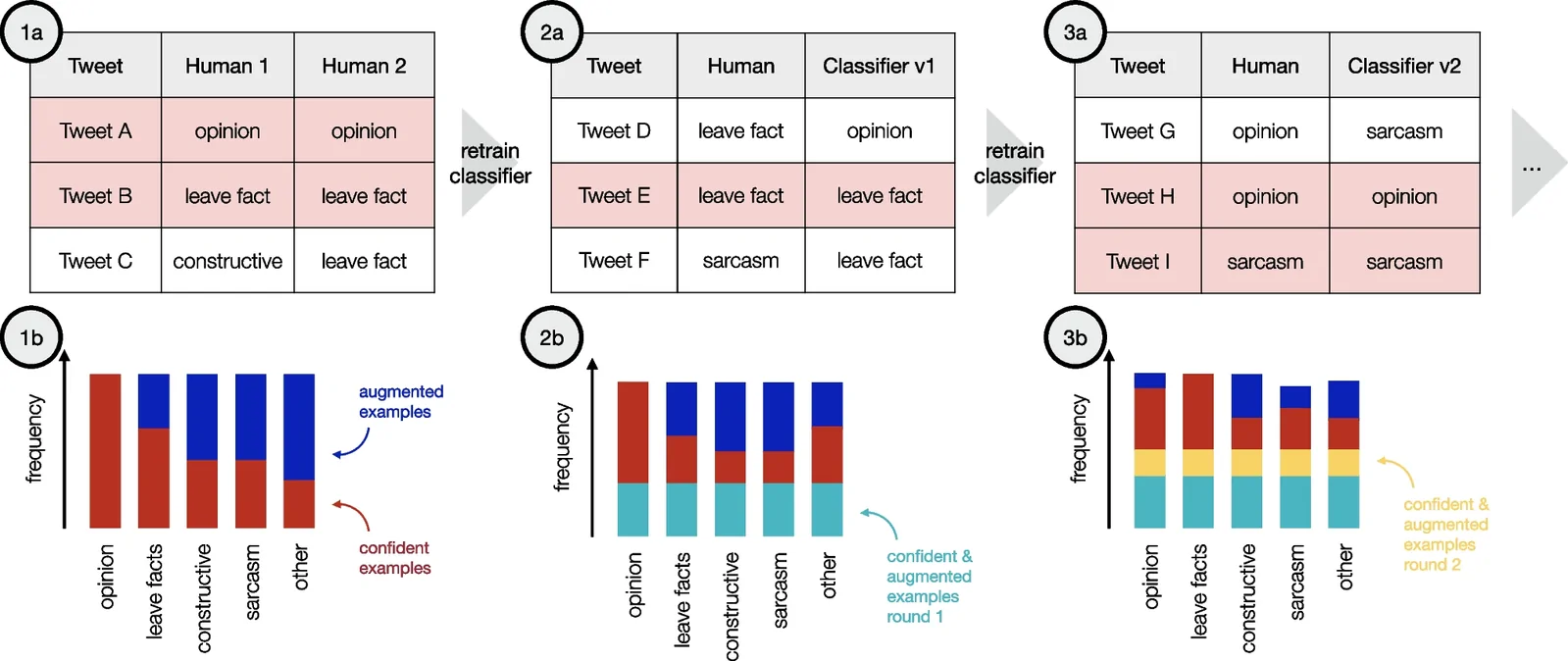
Training on confident examples. *Note.* “Training on Confident Examples” is a training strategy leveraging the most informative samples from the human-annotated training data for classifier training. First, confident examples are identified as annotated data where two or more humans agree (1a), which are then supplemented by augmented examples to counter class imbalance (1b). Second, confident examples are identified as samples with agreement between one human and a preliminary classifier (2a), again supplemented by augmented examples (2b). This process can be repeated multiple times (3a and 3b) or until classifier performance no longer improves. Constructive = constructive comments, leave fact = leaving factual discussion

#### Focus model performance: training on confident examples

To label our training data, we used trained experts (see section “Composing the annotation team”) and most observations of our sample (n=14,282) were labeled by a single annotator. Out of this set, 2242 observations had two labels. These were the tweets that were either included in the first labeled batch that was used for classification scheme development (see the section “Developing a valid classification scheme”) or that were labeled by two annotators to keep track of interrater agreement throughout the annotation process. The IRR pointed towards substantial ambiguity when it came to assigning a single label to a given tweet, even though we reached or exceeded levels of IRR for similar tasks known from the literature (Cheng, Danescu-Niculescu-Mizil, & Leskovec, 2015; Wulczyn, Thain, & Dixon, 2017). For example, out of the observations for argumentation strategy that had two labels, annotators agreed on the label in only 57% of the cases. We call these observations where two or more annotators agree on “confident examples.” Using data annotated by a single annotator during training introduced many confusing [text, label] pairs that impaired classifier learning.

In addition to the ambiguity of the annotation task, and even after our efforts to bias the selection of examples included in the training data towards minority classes (see the section “Focus unbalanced training data: iterative annotation”), classes in the training data were still severely imbalanced with a ratio of 3:1 between the most and least frequent class. This made fine-tuning of the machine learning model challenging, as the classifiers were prone to show significantly worse performance for classes that had a low number of training examples. A way to address class imbalance is the use of data augmentation. The goal of data augmentation is to create more examples of classes that are underrepresented in the training data by creating variations of existing examples that have the same meaning but different phrasing.

For our project, we made use of a data augmentation strategy called “back-translation” (Sennrich, Haddow, & Birch, 2016; Shorten, Khoshgoftaar, & Furht, 2021), where a given text in some language is translated into another language and then back to the original language, frequently leading to a rephrasing of the text, for example via replacing individual words with synonyms. We used MarianMT models (Junczys-Dowmunt et al., 2018) to translate each confident example into 17 target languages and back to German. We removed all direct translation duplicates and then calculated the cosine similarity between the translation and the original text. Examples with a similarity in the bottom and top 10$^\textrm{th}$ percentile were discarded to get rid of examples that were either too similar, adding no new information for the classifier, or too dissimilar, indicating a failed translation. For each minority class, we then added augmented examples generated via back-translation to the training data set until we ran out of augmented examples or there were as many examples of the minority classes as of the most frequent class. Today, instead of back-translation, a common augmentation technique is to prompt LLMs to reformulate the examples in the training data.

Next to balancing the number of examples from different classes in the training data, we also wanted to make use of as much of the available human-annotated data as possible to improve the ability of the classifier to generalize beyond the training data set. To make use of the examples that only had a single human label, we followed an iterative training strategy where we trained the classifiers in several stages, using increasing amounts of annotated data where annotators agreed on the label, supplemented with augmented examples; and where annotators agreed with labels inferred by preliminary versions of the classifier, again supplemented with augmented examples.

We started with a classifier trained only on confident human-annotated examples and supplemented with augmented examples (as described above). We then used this classifier to infer labels for the examples that had only a single label from a human annotator. Using this inferred label as a second label, we then created new confident examples where the human annotator and the (preliminary) machine learning classifier agreed. We added these new examples to the classifier’s training data, ensuring a balanced distribution by supplementing augmented data and adding only the minimum number of new examples for each class. For classes where we had more new examples available than needed, we selected the examples to add at random.

We then re-trained the classifier using the new training data set and again inferred labels in the remaining examples that had only one label from a human annotator. We repeated this process for a maximum of two times or until the performance of classifiers did not increase anymore (i.e., decreased in the consecutive training iteration). It is possible to perform this strategy over more than two iterations.

Figure 5 visually represents the different stages of “Training on Confident Examples”.

## Statistical analysis

### Accounting for classifier inaccuracy

#### Purpose and challenges

After model training, we can run the final classifier(s) over the entire corpus to quantify the variables needed for the statistical analyses. However, even for classifiers with high discriminatory ability, some residual classification error remains. These errors are quantified by metrics such as the F1 score or confusion matrix (see Appendix C for details) and can stem from flawed training data, human annotation, or simply from the ambiguity of the concept.

Variables quantified through machine learning can potentially bias the results of the downstream statistical analyses (Egami, Hinck, Stewart, & Wei, 2023). Although classification error can result in both type I and type II errors, it is almost never reflected or corrected for (TeBlunthuis, Hase, & Chan, 2024). Here, we review best practices for correcting classification errors in regression analysis. We further propose new approaches for error correction, when comparing between groups (e.g., matching; see the section “Focus group comparisons”).

#### Best practices

Correction methods for classification error can be divided according to their purpose: some are applicable to independent versus dependent variables, some correct for unsystematic versus systematic errors, some are more generally applicable to all scenarios (Egami et al., 2023; Teblunthuis et al., 2024).

Regression calibration (Fong & Tyler, 2021) corrects for unsystematic errors in independent variables. It regresses the labels obtained through the classifier on the human annotations from the validation set and all other independent variables, and uses those predictions in the statistical analysis.

Multiple overimputation (Blackwell, Honacker, & King, 2024) corrects for unsystematic errors and systematic errors in the dependent variable. The method bootstraps several parallel data sets, where the labels for the classified variables are drawn based on the likelihood of the true label (again inferred from the validation set) given the automated label and other independent variables. Statistics are computed for each of the parallel data sets and combined.

Pseudo-likelihood modeling (Zhang, 2021) corrects for unsystematic errors. It computes the likelihood of the classified variable given its automated labels, precision and recall of the classifier (i.e., misclassification rates), and the parameters of the regression model. The regression parameters are set to maximize this likelihood.

Maximum likelihood adjustment (Carroll, Ruppert, Stefanski, & Crainiceanu, 2006; Teblunthuis et al., 2024) is suitable for both dependent and independent variables, as well as systematic and non-systematic errors. It estimates the likelihood of the observed data given the true labels (which in turn depend on the classification error), all other independent variables, and the parameters of the regression model. The parameters are again set to maximize this likelihood.

Egami et al. (2023) proposed design-based supervised learning (DSL), a method that corrects labels produced by machine learning classifiers to preserve key statistical properties, such as valid 95% confidence interval coverage, in downstream analyses. Essentially, DSL uses human-annotated gold standard labels, predictions of classifier labels based on the independent variables and the human-annotated data, and the probability of a correct classifier label given the independent variables, to create corrected classifier labels. Those “pseudo-labels” are then employed in the statistical analyses. DSL is available as an R package (Egami, Hinck, Stewart, & Wei, 2025).

Finally, prediction-powered inference (PPI; Angelopoulos, Bates, Fannjiang, Jordan, & Zrnic, 2023) uses human-annotated gold standard data to compute a corrective value to add to the population statistic (e.g., the population mean, or a regression coefficient) that was calculated with inferred labels from a machine learning classifier. The framework is applicable to many statistics and agnostic to the applied classifier. It guarantees reliable point estimates and narrow confidence intervals compared to estimates on the human-annotated data or predictions alone. PPI is available as a Python package (Angelopoulos, Bates, Fannjiang, Jordan, & Zrnic, 2025).

Although suitable for regression tasks, those methods are not applicable to cases of group comparisons, where other questions become relevant: How do we divide our data into two groups that are maximally distinct on the relevant dimension (see the section “Focus group comparisons: binarizing classes”)? And how do we mitigate the classification error in the group comparison (see the section “Focus group comparisons: classifier based bootstrapping”)?

#### Focus group comparisons: binarizing classes

In our working example, one question was the effectiveness of different argumentation strategies against subsequent hate in online discussions. We identified instances where a tweet of user A was replied to by user B, again followed by a tweet of user A. We compared groups of argumentation strategies by user B (e.g., “opinion” versus “not opinion”) and measured hate in tweets of user A, examining whether there was a reduction or increase in hate after said argumentation strategy was used by B. To perform our analysis of choice, non-parametric matching (Stuart, 2010), we needed to distinguish between tweets containing or not containing a specific argumentation strategy. However, our approach can be applied to any setting in which two or even more groups are compared (e.g., *t* test, chi-squared test) and in which groups are not randomly assigned, but identified through classifier labels.

For each tweet of user B, we needed to decide whether it contained a certain argumentation strategy or not. This equals to finding a probability returned by the machine learning classifier above which a tweet would be labeled as a given class (classification threshold). Since we did not randomize people into groups and instructed them to use a certain strategy, the question arises of how to divide the data into groups such that they are maximally distinct. That is, when solely relying on the maximum probabilities provided by the classifier to assign samples to classes, we would end up labeling tweets as opinion, where the classifier is actually not very confident (e.g., the probability for “opinion” is 0.35, but the probability for “sarcasm” is 0.34). To avoid those cases, we will tune the classifier thresholds against the human-annotated data.

First, for probability thresholds incrementing in steps of 0.01, we binarized the argumentation strategies (e.g., “opinion” versus “not opinion”) and computed F1 scores on the human-annotated validation set for each threshold. In other words, we tested a range of classification thresholds to determine the best classifier accuracy. We then chose the threshold with the highest F1 score, maximizing the separation between the two groups. Table 4 illustrates our approach with a toy example.

**Table 4:** Tuning classifier thresholds against human annotations for group comparisons

Tweet	Human	Classifier probability	Threshold 0.73	Threshold 0.74
Tweet A	opinion	.742	opinion	opinion
Tweet B	not opinion	.737	opinion	not opinion
Tweet C	opinion	.741	opinion	opinion
Tweet D	opinion	.739	opinion	not opinion
Tweet E	not opinion	.725	not opinion	not opinion
Tweet F	opinion	.749	opinion	opinion
Tweet G	not opinion	.741	opinion	opinion
Tweet H	not opinion	.722	not opinion	not opinion
**F1 score**			**.8**	.75

*Note.* For each group comparison, we incremented classifier thresholds by 0.01, computing F1 scores for each of those thresholds according to the human-annotated validation set. Here, only two out of many tested thresholds are illustrated. To separate the data sets into groups, we chose the threshold maximizing the F1 score, here 0.73 for argumentation strategy “opinion” (toy example)

**Table 5:** Bootstrapping confidence intervals for group comparison based on false-positive/negative rates

Tweet	Classifier Label	Sample 1	Sample 2	Sample 3
Tweet A	opinion	** not opinion**	opinion	opinion
Tweet B	not opinion	**opinion**	not opinion	not opinion
Tweet C	opinion	opinion	opinion	**not opinion**
Tweet D	opinion	opinion	**not opinion**	opinion
Tweet E	not opinion	not opinion	**opinion**	**opinion**
Tweet F	opinion	opinion	opinion	opinion
Tweet G	not opinion	not opinion	not opinion	**opinion**
Tweet H	not opinion	**opinion**	**opinion**	not opinion
Regression coefficient		.45	.49	.37
Confidence interval			[.37 - .49]	

*Note.* We repeated our matching analysis on several samples, for which we flipped labels according to the false negative rates (“opinion” to “not opinion”) and false positive rates (“not opinion” to “opinion”) of our classifier. Here, flipped labels are marked in bold. For each sample, we compute coefficients to obtain an overall confidence interval across all samples. The approach makes the group comparison robust to the errors of the classifier

For measures with more than two classes, we first created one hot encoded variables labeling a tweet as 1 if a given strategy was present and 0 if not. Importantly, this approach cannot be applied when validating the classifier since tuning thresholds against the human annotations inflates performance metrics. For validation purposes (see the section “Evaluating model performance”), we used the maximum probability returned by the classifier to label the tweets.

#### Focus group comparisons: classifier based bootstrapping

While tuning the classifier threshold to human annotations ensures the classes in the group comparison are as distinct as possible, the problem of classifier inaccuracy persists. To solve this problem and following an approach similar to Card et al. (2022), we calculate the rate of false positives and false negatives for each category of a classifier. We then use these rates to resample a simulated treatment variable as part of generating bootstrap samples used to calculate confidence intervals.

To illustrate: We compared tweets where a user applied giving an “opinion” versus tweets with “no opinion”. If the false-negative rate for the classifier on opinion was 0.125, we flipped the label “opinion” to “no opinion” in 12.5% of the cases. Likewise, if the false-positive rate was 0.25, we flipped the label “no opinion” to “opinion” in 25% of the cases. This way, we drew multiple samples with replacement, repeating the matching analysis, effectively computing confidence intervals for our estimates that are inflated and therefore robust to the noise introduced by the machine learning classifier. Table 5 illustrates our sampling approach.

## Discussion and outlook

Here, we have discussed best practices for computational social mixed methods pipelines combining social science techniques and machine learning, including data annotation, model training, and statistical analysis (Fig. 1). We exemplified the pipeline with a working example, in which we investigated the effectiveness of counter speech against online hate. Specifically, we introduced solutions for common obstacles of such approaches. In “Focus unbalanced training data: iterative annotation”, we introduced “Iterative Annotation” to balance the training data over the classes that the classifier is supposed to infer to improve classifier performance. In “Focus model performance”, we introduced “Training on Confident Examples” as a way to leverage the most informative human-annotated data for model training to improve performance. Finally, in the section “Focus group comparisons”, we proposed error mitigation mechanisms making the statistical analyses robust to the uncertainty of the machine learning classifiers in group comparisons. Furthermore, we added to the discussion of when LLMs are suitable classification models, providing our own experiment comparing LLMs to customized transformer models for a classification task from our working example.

We presented alternatives and best practices to provide an overview of the entire pipeline from start to finish, and discussed how decisions made at some point can affect upstream or downstream tasks. That said, exploring all potential choices is out of scope for any single tutorial, and other viable alternatives exist that we did not discuss. For example, we decided that each observation in our data is uniquely assigned to one class (multi-class classification; Grandini, Bagli, & Visani, 2020). However, the possibility exists for each of the observations to be assigned to more than one class (multi-label classification; Tsoumakas & Katakis, 2007). Furthermore, while we used “Training on Confident Examples” (see section “Focus model performance”) to disambiguate disagreement between annotators, other approaches such as multi-annotator architectures exist which preserve and model the internal consistency in each annotator’s labels (Davani, Díaz, & Prabhakaran, 2022). Multi-annotator architectures include ensemble learning (Polikar, 2012), multi-task learning (Liu, He, Chen, & Gao, 2019), and multi-label learning (Zhang & Zhou, 2006), for which we give additional details in Appendix E.

Large, longitudinal textual corpora that are now more often available to social scientists provide, in principle, an opportunity to investigate relationships between different cognitive-social phenomena. Those phenomena are often complex, that is, they are latent with no single, objective possibility for conceptualization and operationalization. Our guidelines provide solutions to challenges that arise when assessing complex constructs in large-scale text. Examples include handling unbalanced observations in data not primarily designed for research (see section “Focus unbalanced training data: iterative annotation”), mitigating interrater reliability through “Training on confident examples” (see section “Focus model performance”), and accounting for classification inaccuracies in the statistical analyses (see sections “Focus group comparisons: binarizing classes” and “Focus group comparisons: classifier based bootstrapping”). While large-scale text data can provide new, interesting perspectives on social science questions, inferences from observational data can be influenced by many confounding factors even after following best practices in data annotation, model training, and statistical analysis (Rosenbaum, 2002). The nature of these biases will depend on the particular textual corpus and the research question. For example, in our working example, local social norms and broader societal institutions affect behaviors over and above beliefs and intentions expressed in text (Álvarez-Benjumea & Winter, 2020). Consequently, when scale is not the primary concern, researchers might choose different analytical paths, such as in-depth qualitative analysis, to uncover more nuanced relationships between the phenomena of interest (Teblunthuis et al., 2024).

As evident from the considerations above, using machine learning for social science research involves many steps and choices. In this context, reproducibility of results becomes an issue, as each choice can substantially influence the outcome of the research process. Similar to the social sciences, the machine learning community has recently started to discuss difficulties in the replication of results (Kapoor & Narayanan, 2023; Semmelrock, Kopeinik, Theiler, Ross-Hellauer, & Kowald 2023). Machine learning models do not learn independently (practitioners still need to make a considerable amount of choices such as setting the loss function; see also Brooks, 2024), and patterns extracted by models are not automatically meaningful (on the contrary, they might have none or multiple interpretations with respect to human perception; Heuer, Jarke, & Breiter, 2021). Therefore, choices should be documented, and intermediate research outcomes should be made available wherever possible. This need has been recognized by the machine learning community, resulting in the formulation of a number of reporting standards for human annotation (Geiger et al., 2021), data (Gebru et al., 2021), and models (Mitchell et al., 2019), as well as overall reporting recommendations (Kapoor et al., 2024; Parsons et al., 2022), which we discuss in Appendix F.

Although laid out in three consecutive steps, mixed methods approaches are seldom linear. We often explored different paths in parallel (e.g., different forms of data augmentation). Moreover, making modeling decisions at certain points in the process can only be informed by the knowledge accumulated so far. For example, because we deemed interrater reliability sufficient, we decided to acquire only one annotation for the majority of tweets in the training data – a decision that later turned out to be suboptimal. There is also the possibility that some steps only improve little (e.g., hyperparameter tuning), whereas others change a lot (e.g., “Training on Confident Examples”). However, it is hard to tell at the beginning which approach introduces the biggest performance gain. In general, data, theory, and machine learning methods can inform each other in various ways. For example, we can use machine learning for exploratory data analysis to inform theory building. On the other hand, we can adapt our machine learning models to theory (e.g., by selecting theoretically relevant classes), enabling us to test existing hypotheses at scale.

Despite their advantages, well-rounded computational social-mixed-methods pipelines remain scarce, which may be due to several challenges they must face. First, the process from developing a classification scheme from scratch to training working classifiers can be lengthy, clashing with the fast-paced publication environment, especially in machine learning-based research. Second, mixed-methods approaches include a plethora of techniques that can be hard to master, especially across disciplinary boundaries (Malik, 2020). That is true for traditional qualitative methods such as Grounded Theory, as well as for quantitative methods such as statistical modeling or machine learning classifiers. Third, qualitative methods have (maybe unfairly) been criticized for their lack of characteristics important to quantitative researchers, such as standardization (Bhati, Hoyt, & Huffman, 2014). In that sense, combining the small-scale manual analysis of text with machine learning needs to bridge large cultural gaps. Fourth, creating data sets is traditionally valued less compared to performing statistical analyses (Jarrahi, Memariani, & Guha, 2023), although this is starting to change (Gebru et al., 2021). Still, computational social mixed methods pipelines can be seen as a modern form of methodological triangulation (specifically triangulation of methods, investigators and data sources; Rothenbauer, 2008), which makes them especially valuable.

Moving forward, efforts have to continue to translate practices and terminology between the fields involved in computational social science. Interdisciplinary collaboration can also be an opportunity to challenge standards within a discipline, such as valuing predictive performance over construct validity (Baden et al., 2022), or evaluating data quality against unattainable standards for interrater reliability in the wild (Landis & Koch, 1977). While computational scientists need to learn about the rich history of theory and methods in the social sciences, social scientists need to keep up with the rapid development in machine learning. Our guidelines for rigorous mixed-methods approaches blending social science and computational techniques demonstrate that each field can and should make valuable contributions to fully exploit their potential for fostering understanding of social science phenomena.

## Figures and Tables

**Fig. 6:**
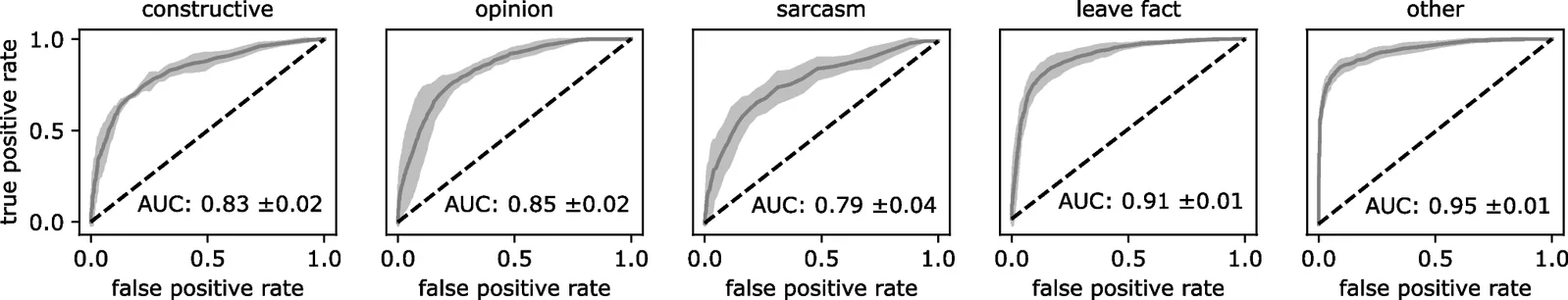
Receiver operating characteristic of a machine learning classifier predicting the five argumentation strategy classes. *Note*. Receiver operating characteristic (ROC) curves and area under the curve (AUC) are averaged over the performance of each of the five models trained on five different data splits. *Shaded areas* indicate standard deviations

**Fig. 7:**
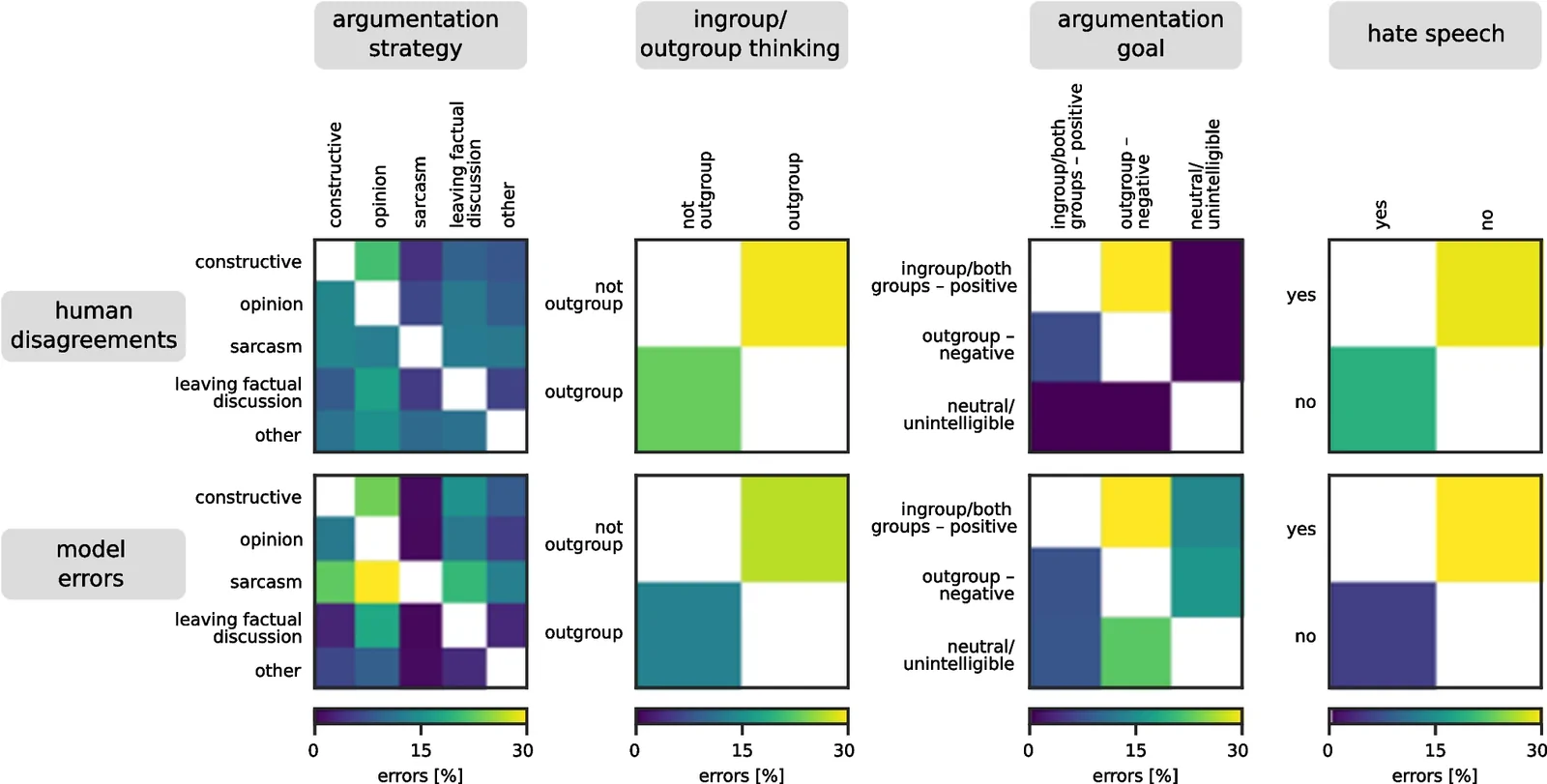
Human disagreements and model errors. *Note*. For human disagreements, the percentage indicates the number of instances in which an observation was annotated as two different classes, divided by the number of cases in which annotators agreed on the label. For model errors, the percentage indicates the number of instances in which the model predicted a class different from the class in the test set

## Data Availability

We provide sample data from our working example to run the code in our repository. We used GPT-4.1 mini to rephrase the tweet text to abide to the Twitter terms of service, not allowing us to publish the raw tweets.

## Appendix A: GPT-4 prompts

**Zero Shot w/CB** You are a data labeler, here are the labels and instructions of each label:

’0: inclusionary about in/both groups’; which is assigned if the text highlights positive characteristics of the ingroup or justifies their actions, or if the text is pointing out common characteristics or challenges of in- and outgroup

’1: exclusionary about outgroup’; which is assigned if the text points out realistic or unrealistic threats from the outgroup, or if the text makes members of the outgroup look stupid

’2: other’; which is assigned if the text does not have in- or outgroup thinking, or if the text has signs of in- or outgroup thinking, but the speaker’s affiliation is not apparent. Using these labels and instructions please label the following text: {out-of-sample}

**Few Shot w/o CB** You are a data labeler, here are the labels:

0: inclusionary about in or both groups

1: exclusionary about out-group

2: other Here are a few examples:

{example0} This is labeled as 0: inclusionary about in or both groups

{example1} This is labeled as 1: exclusionary about out-group

{example2} This is labeled as 2: other Please label this message: {out-of-sample}

**Few Shot w/ CB** You are a data labeler, here are the labels and instructions of each label:

’0: inclusionary about in/both groups’; which is assigned if the text highlights positive characteristics of the ingroup or justifies their actions, or if the text is pointing out common characteristics or challenges of in- and outgroup

’1: exclusionary about outgroup’; which is assigned if the text points out realistic or unrealistic threats from the outgroup, or if the text makes members of the outgroup look stupid

’2: other’; which is assigned if the text does not have in- or outgroup thinking, or if the text has signs of in- or outgroup thinking, but the speaker’s affiliation is not apparent. Here are a few examples:

{example0} This is labeled as 0: inclusionary about in or both groups

{example1} This is labeled as 1: exclusionary about out-group

{example2} This is labeled as 2: other Using these labels, instructions and examples please label the following text: {out-of-sample}

## Appendix B: Cost estimation of GPT-4 as an annotator or classifier

In terms of budget, for the GPT-4 based labeling we outlined in the main, we estimate each label to cost $0.0086 on average (as of April 16, 2024), resulting in $135 to label the entire training set used in our study (n=15,692) on one dimension and $540 on all four of our dimensions of interest (argumentation strategy, in- and out-group content (two step procedure), hate speech). Costs can vary with the length of the prompts and the version of the model being used. However, assuming that researchers will opt for the most recent model, and considering the fact that so-called legacy models (like GPT-4 in March 2026) can be more expensive than the most recent ones, this cost estimate remains a somewhat accurate guide for labeling costs with LLMs (for more details, see https://developers.openai.com/api/docs/pricing/). On the other hand, the annotation team consisting of four annotators was paid $9,205 in total ($0.58 per label), which includes the development and refinement of the classification scheme—an essential task that GPT-4 cannot do. Labeling the whole data set (n=1,150,469) with GPT-4 on all four dimensions would cost $39,576 based on our estimate above, whereas the training of a customized classifier (or the application of an open-source or open-weights LLM like Meta’s Llama or Kimi’s K2.5) necessitates acquiring computational resources, whether that be physical computers or cloud computing, as well as the cost of labeling the training data.

## Appendix C: Assessing model performance: performance indicators

The output of a machine learning model trained for a classification task is a probability that a given example belongs to each class. In multi-class classification, a softmax function produces class probabilities that sum to 1; in multi-label classification, a sigmoid function predicts the presence or absence of each label separately. The probabilities are then transformed into binary class labels by either selecting the class with the highest probability (multi-class classification) or applying a threshold above which an example is assigned to a class (multi-label classification). When considering these binary predictions, there are four possible outcomes: the model correctly predicts the presence of a class (true positive), the model correctly predicts the absence of a class (true negative), the model wrongly predicts the presence of a class (false positive), and the model wrongly predicts the absence of a class (false negative). Several metrics exist that combine these outcomes in various ways, and depending on the nature of the classification task, different metrics are more suitable to measure model performance.

### C.1: Accuracy

A standard metric is the *accuracy* of model predictions, which simply measures the fraction of correctly classified examples (e.g., the sum of true positives and negatives over the total number of examples). Accuracy, however, is not a very good performance metric when classes are severely imbalanced. To illustrate this point, in an extreme case where 95% of instances belong to one class, a classifier that always predicts the majority class would achieve an accuracy of 0.95 (with 1.00 being the achievable maximum).

### C.2: F1 score

A metric that is better suited to assess model performance when classes are imbalanced is the F1 score, the harmonic mean between precision and recall of the model. Precision is defined as the number of true positives divided by the sum of true and false positives. Recall (also called “sensitivity”) is the number of true positives divided by the sum of true positives and false negatives.

While accuracy is easy to calculate for classification problems with more than two classes, the *F1 score* of a multi-class prediction task is not as straightforward to calculate. In a first step, the performance of the model is assessed for each class individually by calculating the F1 score for every class in a one-vs-rest scenario (i.e., one class against all the others). The overall performance of the classifier for all classes combined can then be assessed by combining the F1 scores of all classes. There are several ways to combine F1 scores: the arithmetic mean of F1 scores (*macro average*), the mean of F1 scores weighted by their “support” (*weighted F1*), i.e., the number of examples of a given class in the test data set, and the *micro average* that is calculated by first counting the global number of all true positives, false positives and false negatives, calculating the global precision and recall and then calculating the F1 score as their harmonic mean. For multi-class classification, the micro-averaged F1 score is the same as the accuracy, since the proportion of correctly classified observations is computed. In the case of an imbalanced data set where all classes have equal importance, the macro-averaged F1 score is a sensible choice, since it gives each class equal weight, independent of its frequency in the test data set. This is also the metric we chose to assess the performance of our classifier.

### C.3: ROC & AUC

An alternative way to assess the performance of a classifier is to assess its *receiver-operating characteristic* curve (ROC curve; Hanley & McNei, 1982). ROC curves are calculated by comparing predicted class probabilities for a single class in a one-vs-rest scenario to gold standard labels. To assign a label to an observation, a threshold on the predicted class probabilities is introduced, and observations with a probability above the threshold are assigned the corresponding class label. The ROC curve is the plot of the true positive rate over the false positive rate as the threshold is varied from 0 to 1. Exemplary ROC curves for the five argumentation strategy classes are shown in Fig. 6.

The farther up and left the curve stretches, the better the classifier is at discerning true positive instances and true negatives of a class. To summarize the performance of the classifier in a single number, the area under the curve (AUC) is calculated. An AUC of 0.5 would be equal to the diagonal line and signifies that the classification performance is no better than chance. AUC values between 0.8 and 0.9 are typically considered as “excellent discrimination” while values between 0.7 and 0.8 are still considered as “acceptable discrimination” (Hosmer, Lemeshow, & Sturdivant, 2013).

### C.4: Confusion matrix

Lastly, independent from metrics used to summarize model performance, it is always a good idea to study the *confusion matrix* of a classifier. The confusion matrix shows which classes the model tends to confuse with other classes (false positives). The confusion matrix is a square table (or heat map) with predicted values by the classifier as rows and actual values as established by human annotation as columns (or vice versa). For our project, we show the confusion matrices for all four classifiers we trained in Fig. 7 (bottom row). Since we also have examples with more than one label from a human annotator, we can identify the class pairs for which humans tend to disagree the most (also shown in Fig. 7, top row). If the machine learning classifier tends to reproduce human errors, this supports the assumption that by applying the classifier to automatically label data, no biases that are different from human biases will be introduced.

## Appendix D: Model training: hyperparameters

### D.1: Batch size

Batch size refers to the number of training examples used in one iteration or step of training. During each iteration, the model processes a batch of data, calculates the loss or error, and updates its parameters to improve performance. This process is repeated for multiple iterations until the model converges (i.e., the loss stops decreasing) or reaches a set number of iterations. A larger batch size means the model sees more examples in each iteration, which can speed up training but requires more computational memory (VRAM). A smaller batch size means the model learns from fewer examples at once, which can slow down training but requires less computational memory.

### D.2: Learning rate

The learning rate determines how quickly or slowly a model adjusts its weights based on errors during training. Weights are numerical parameters that are learned by the model during the training process. A very high learning rate may make the model perform poorly and a very small value may result in a longer time to learn or even failure to learn the target task. Finding an optimal learning rate is essential for the model to learn effectively and perform well.

### D.3: Warm-up steps

Warm-up steps are the number of steps at the beginning of training where the learning rate gradually increases, allowing the model to slowly adjust to the training data. This gradual increase helps the model explore the training space more effectively without making large and sudden changes to the model weights. This is important when fine-tuning a pre-trained model.

### D.4: Label smoothing

Label smoothing is a technique used to make the training process more robust and prevent the model from becoming overconfident in its predictions. It involves adjusting the target labels used during training by a value so that instead of aiming for a probability of 1 for the correct label and 0 for all others, the model distributes its probability mass across all possible labels. In this way, the model is a bit less certain about its predictions during training, which can help it learn more effectively and make better decisions when faced with new examples.

### D.5: Weight decay

Weight decay is a technique used during training to prevent the model from becoming overly specialized and to help it generalize better to unseen data. The magnitude of the weights can influence the model’s behavior. Larger weights can lead to more complex models that may overfit the training data, while smaller weights can lead to simpler models that may underfit the data. Controlling the magnitude of weights helps regularize the model and prevent overfitting or underfitting by avoiding excessively large or small weights. This ensures that models generalize better to unseen data and improve overall performance.

## Appendix E: Details on alternative model architectures

Ensemble learning (Polikar, 2012), i.e., training an ensemble of models, each on the labels provided by a single annotator, has successfully been used to model annotator perspectives and polarized opinions to improve hate speech detection (Akhtar, Basile, & Patti, 2020). Predictions from the ensemble of models can then be used either in a disaggregated form, allowing multiple labels per observation, or aggregated, for example, via majority voting. A downside of ensemble learning is that it requires enough examples from each annotator to train a separate model realistically, and that training multiple separate models can be computationally expensive.

Another approach is offered by multi-task learning (Liu et al., 2019) where a single model learns to perform several tasks simultaneously, instead of just one. In the context of learning a classification task based on data from different annotators, the model learns multiple annotators’ perspectives as separate classification tasks. In contrast to ensemble learning, the different tasks are not isolated and share encoder layers to generate a representation of the input sequence. Differentiation between tasks happens in the final classification layer: the model has a separate classification head for each annotator. Since only the classification head is trained separately for each annotator, this approach reduces computational cost when compared to ensemble learning. As an additional benefit, since the different tasks share a common model core, learning each task can help the model understand the other tasks.

Lastly, multi-label classification uses the same model architecture as multi-class classification. However, in the multi-label problem, the labels are nonexclusive, i.e., the sum of label probabilities outputted by the model can be larger than one. In practice, this works by either transforming the classification problem into a series of binary- or multi-class classification problems, or adapting the model’s back-propagation algorithm to accommodate multi-label learning (Zhang & Zhou, 2006). This approach modifies the training process and not the model architecture. Commonly used training libraries such as Hugging Face’s transformers (Wolf et al., 2019) offer functionality to implement such training approaches (Vasylevskyi, 2024).

## Appendix F: Details on reporting standards

### F.1: Data reporting

After a lack of data documentation led to issues including discrimination and lack of reproducibility in machine learning, researchers called for data reporting standards to alleviate those risks. Today, datasheets (Gebru et al., 2021) are a common form to report data sets, which machine learning models are trained on. Gebru et al. (2021) provides 57 questions that tease information relevant to the reporting of data sets in seven categories: (1) motivation, (2) composition, (3) collection process, (4) preprocessing, cleaning, and labeling, (5) uses, (6) distribution, and (7) maintenance. There are alternative or complementary approaches like data statements for natural language processing (Bender & Friedman, 2018) or data nutrition labels for automated decision-making systems (Chmielinski et al., 2022). In general, social scientists have already established strong data reporting standards, but the questions can help to ensure completeness for machine learning applications, a method that is new to the field.

As outlined in our guidelines, a common data source in natural language processing is the labeling of text data by human annotators. Although there is no reporting standard for human-annotated data in particular, studies have examined the reporting of a range of quality criteria in human-annotated data for machine learning and therefore give implicit recommendations on which information should be included. Geiger et al. (2020, 2021) evaluate the reporting of human annotated data on the following dimensions: who the annotators were, the number of annotators, whether formal instructions were given to annotators and made transparent, how the annotators were trained, the calculation of interrater agreement, the compensation of annotators, and – for crowd-working platforms – quality checks.

### F.2: Model reporting

The equivalent of datasheets (Gebru et al., 2021) for data sets are model cards (Mitchell et al., 2019) for machine learning models. Model cards are short summaries for trained models, ensuring the adequate use of those models. Model cards include eight subsections: (1) model details (e.g., type of model), (2) intended use, (3) factors (i.e., relevant subgroups like gender in the training data), (4) metrics (e.g., performance measures), (5) training and evaluation data, (6) quantitative analyses with respect to the factors mentioned above, (7) ethical considerations (e.g., sensitive data in the training data), and (8) caveats and recommendations including everything that did not go into the other sections. Mitchell et al. (2019) provides exemplary model cards.

### F.3: Overall reporting recommendations

Across different fields, common mistakes in the application of machine learning have been identified. The REFORMS checklist (Kapoor et al., 2024) lists recommendations for machine learning-based science, which is defined as research employing machine learning to arrive at scientific claims, as opposed to machine learning being the subject of investigation itself. The checklist includes 32 questions in eight categories: (1) study goals, (2) computational reproducibility, (3) data quality, (4) data preprocessing, (5) modeling, (6) data leakage, (7) metrics and uncertainty, and (8) generalizability and limitations. Beyond reporting standards, REFORMS can already be applied during the research, offering guidance on how activities should be conducted to adhere to state-of-the-art practices. Many of those practices are also discussed in our guidelines. The checklist helps authors and reviewers alike to navigate a piece of research and judge its quality.

Another movement relevant to the reporting of scientific work is the Open Science movement. Open Science is the idea that scientific knowledge should be accessible, transparent, rigorous, reproducible, replicable, accumulative, and inclusive (Parsons et al., 2022). It commonly entails practices such as open data, open methodology, and open source. Similar to reporting standards, Open Science practices aim to improve the quality of scientific outcomes. The FORRT glossary (Framework for Open and Reproducible Research Training, https://forrt.org/glossary) provides an extensive collection of Open Science practices and terms.

## Code Availability

Commented, tutorial-style code is publicly available on GitHub under https://github.com/Hai-Lina/computational-social-mixed-methods-pipelines.
